# Linking microbial taxonomy and function in N and P metabolism: a study of organic amendments in semiarid restored soils

**DOI:** 10.1186/s40793-025-00845-9

**Published:** 2026-01-08

**Authors:** Ana B. Villafuerte, André M. Comeau, Rocío Soria, Raúl Ortega, Robyn J. Wright, Isabel Miralles

**Affiliations:** 1 Department of Agronomy & Centre for Intensive Mediterranean Agrosystems and Agri Food Biotechnology (CIAIMBITAL), University of Almería, E-04120 Almeria, Spain; 2 Integrated Microbiome Resource (IMR), Dalhousie University, Halifax, Nova Scotia Canada; 3 School of Advanced Agricultural and Forestry Engineering and Biotechnology, Campus Universitario s/n, Castilla La Mancha University, E-02071 Albacete, Spain

**Keywords:** Nitrogen metabolism, Phosphorus turnover, Soil restoration, Bacterial taxonomy, Shotgun metagenomics, Functional profile, Degraded quarry soil

## Abstract

**Background:**

Arid and semi-arid regions cover approximately 41% of Earth’s surface and their soils are highly vulnerable to degradation due to harsh climatic conditions and extractive activities, such as opencast mining. Organic amendments are widely used to restore degraded soils because they improve physical, chemical, and biological properties. However, little is known about how these amendments alter microbial communities and the relationship between microbial taxonomy and function, particularly in nitrogen and phosphorus cycling. To address this knowledge gap, the effects of different organic amendments (gardening compost, greenhouse horticultural compost, sewage sludge and two blends of the above) on soil properties, microbial communities and their contributions to nitrogen metabolism and phosphorus turnover in degraded soils from a limestone quarry in the Gádor Range (Almería, SE-Spain) six months after their application were investigated.

**Results:**

Organic amendments increased nutrient content (total organic carbon, total nitrogen and available phosphorus), microbiological activity, and bacterial biomass compared to unamended soils, with the largest increases in sewage-sludge-treated soils. Shotgun metagenomic assays revealed that organic amendments modified bacterial community composition and differentially influenced potential function pathways, contributing more strongly to nitrogen metabolism than phosphorus turnover, particularly within the phosphonate pathway. Across soils, *Pseudomonadota* and *Actinomycetota* were the dominant phyla. Sludge-amended soil showed higher relative abundance of *Pseudomonas*, associated with denitrification processes (*nirK*, *nosZ*, *norB*) and phosphonate degradation via C-P lyase (*phnJ)*. Genera such as *Streptomyces* were linked to ammonium assimilation (*glnAd*, *gltBD)* and phosphonate synthesis (*pmmS*), and were more abundant in soil with vegetable-compost and unamended soils. Both nitrogen and phosphorus metabolisms exhibited phylogenetically unrestricted functional patterns, indicating high functional redundancy at phylum and genus levels.

**Conclusions:**

This research establishes key relationships between taxonomy and function in restored soils and demonstrates how organic amendments rephase microbial communities and their potential roles in nutrient cycling. Although dominant taxa and functions were identified, many microorganisms involved in nitrogen and phosphorus turnover remain insufficiently characterized. Further research across restoration contexts is needed to compare nutrient-cycling responses and to deepen understanding of taxonomy-function linkages in soils amended with organic residues.

**Supplementary Information:**

The online version contains supplementary material available at 10.1186/s40793-025-00845-9.

## Background

Arid and semi-arid zones are characterized by low soil nutrients and organic matter, poor soil structure, water limitation, among others, that limit their productivity and fertility [1]. Additionally, these regions are subject to extreme environmental conditions, such as low rainfall, high temperatures and high levels of solar radiation [2], further increasing its vulnerability to degradation [3, 4]. About 41% of the land is covered by drylands and this percentage is expected to increase in the coming years due to climate change and other anthropogenic activities (e.g. open-pit mining) [5]. In these ecosystems, human extractive activities have a drastic effect, causing vegetation degradation [6] and, given the fragility of the landscape due to scarce water resources, also contributing to soil deterioration [7], affecting its ecological functioning [8]. Low nutrient contents limit microbial development and activities with a direct effect on biogeochemical nutrient cycling. This makes these ecosystems more susceptible to further degradation and their natural recovery after disturbance is very slow or in many cases irreversible on a human time scale [9].

The difficulty of recovering these soils has led to the consideration of alternatives such as restoration with organic amendments. Different authors have shown that the incorporation of organic residues in degraded arid and semi-arid soils can improve the physical, chemical, biochemical and biological properties of the soil [10–13]. The application of organic amendments also influences the composition of soil bacterial communities because labile or recalcitrant compounds favor the proliferation of some bacterial communities over others, as well as seeding the soils with allochthonous bacteria [14, 15].

Microorganisms are a key component of the soil biosphere, playing a fundamental role in maintaining soil fertility and functionality by regulating most biological and biogeochemical processes [16]. Their activity directly or indirectly increase the mineralization or solubilization of nutrients such as carbon (C), nitrogen (N) and phosphorus (P), facilitating the transport of essential nutrients to plants as they make the nutrients in the soil more bioavailable [16, 17]. However, these processes are not solely dependent on microbial activity; they are also strongly influenced by soil chemistry, which determines nutrient availability, redox conditions, and the overall efficiency of microbial functions. In turn, the diversity of microorganisms facilitates redox reactions and promotes homeostasis in the soil ecosystem [18]. This intricate relationship between microbial communities and soil chemistry ultimately shapes soil fertility, highlighting the dynamic interplay between biological and chemical factors in sustaining ecosystem productivity [19].

Nitrogen is one of the primary nutrients in terrestrial ecosystems, as well as a key cellular component for the formation of proteins and nucleic acids. Microorganisms are involved in several processes of the nitrogen cycle such as assimilation, ammonification, nitrification, denitrification, anaerobic ammonium oxidation (anammox) and nitrogen fixation [20]. However, these nitrogen-transforming microorganisms can perform various redox functions and form microbial networks that work cooperatively and/or competitively [20]. Most studies on the effects of amendments on nitrogen cycle processes, both at chemical and genetic levels, have focused on agricultural soils [21–23], leaving their impact on non-agricultural ecosystems largely unexplored. In arid and semi-arid regions, nitrogen cycles are characterized by low N availability, minimal atmospheric inputs, and high gaseous losses [24]. Given that organic amendments are rich in nitrogen compounds, their incorporation is expected to influence N pools and processes in these soils.

Another primary element for living organisms is phosphorus; it is an essential part of nucleic acids (DNA and RNA), lipids (phospholipids, fats and oils) and energy storage molecules (ATP) [16]. Microorganisms control the mineralization and solubilization processes of phosphorus and, although this element is abundant in the soil, it is found in insoluble forms and its availability to plants is limited. The conventional phosphorus cycle focusses on the dynamics of phosphates in the environment; however, there are other organic compounds such as phosphinates and phosphonates (C-P bond) and less explored metabolic pathways that can be used by microorganisms as a source of phosphorus to perform a variety of cellular processes [25]. Up until recently, these compounds were thought to be of little environmental importance in terrestrial environments, but some studies now show that biogenic phosphonates play an important and unrecognized role in the P turnover [26–28]. It is known that in the oceans, 25% of the high-molecular-weight dissolved organic P constitutes P-phosphonate [29, 30], however the quantitative importance of biogenic phosphonates in the terrestrial biosphere has not been conclusively determined.

Although it is known that soil microbial communities are of fundamental importance in biogeochemical cycles, and that human activities that remove the most fertile soil layers (such as open-pit mining) directly affect microbial communities, the way in which the application of organic amendments also influences microbial community compositions is a less explored area. Studies of bacterial communities and their diversity have most-often been done with 16 S rRNA gene analysis [14, 15, 31–33], but this technique only natively provides information about the taxa and their relative abundances. However, other culture-independent metagenomic techniques such as high-throughput “shotgun” DNA sequencing have become more popular in the last decade due to improving costs [34]. These techniques enable more comprehensive analysis of microbial communities in different conditions and ecosystems, by providing not only taxonomic information, but also information on the abundance of specific genes and complete functional profiles [35–37]. These new approaches will allow us in the future to better understand soil microbial ecology and the molecular mechanisms of the main ecosystem services provided by “meta-regulating” soils. Therefore, the objective of our work was to analyze the metabolic pathways involved in the biogeochemical cycles of nitrogen and phosphorus and the relationship of taxonomic profiles with the possible functions they play (i.e.: taxonomically-stratified functions) in these cycles in soils restored with organic amendments.

We hypothesized that different organic treatments based on recycled waste from different sources can have a different impact on the physical and chemical properties of the treated soils. Moreover, these changes in soil properties would favor the selective proliferation of different bacterial communities, with possibly some functional redundancy in key N and P functions, but with differing species fulfilling key roles in the biogeochemical cycles. Furthermore, the restoration using organic amendments is expected to induce differential changes in the composition and function of microbial communities compared to control soils, with potential variations depending on the different types of amendments. It is also expected to find different metabolic and functional patterns between the unamended control soils and the natural reference soils (that were not disturbed by mining activity), reflecting the impact of amendments on the restoration of the microbial ecosystem.

## Materials and methods

### Study area

The study was conducted in a limestone quarry in totally degraded soils located in the Sierra de Gádor in Almería (SE, Spain; 36°55′18″ N, 02°30′40″ W, 362 m.a.s.l). The geological material was mainly formed by limestones and dolomites with calcareous sandstones developing mainly into Regosol-type soils [38]. Due to mining activities, the surface has been leveled. According to the nearest meteorological station (weather station from Alhama of Almería), located approximately 4 km from the study site and with similar meteorological characteristics to the study area, the annual average rainfall was 242 mm, with average temperatures of 17.6 °C, reaching 42.7 °C in the summer months (July, August and September) and with average minimum temperatures of 2.8 °C in the winter months (December, January and February) [39]. The resulting substrate after mining activities consisted of a mixture of compacted soils composed mainly of marl and calcareous sandstone rocks where vegetation could not develop naturally [11]. The dominant vegetation in the surrounding area unaffected by mining activities was mainly composed of *Macrochloa tenacissima* (L.) Kunth, *Anthyllis terniflora* (Lag) Pau and *Anthyllis cytisoides* L [39].

### Experimental design and soil sampling

In May 2018, eighteen experimental plots of 10 m x 5 m (50 m^2^), separated by 1 m wide corridors, were laid out at the study site. For a detailed description of the location and distribution of the plots see the work of Soria et al., 2023 [40]. Heavy machinery supplied by the mining company, such as backhoe and bulldozers with rippers that were used for decompaction and homogenization of the substrate at depth of the first 20–30 cm from the topsoil. Using a randomized block design, five treatments (three replicates per treatment) composed of organic amendments made from residues of different origins were homogenized in the first 20 cm of the surface of each plot. The application rates of the organic amendments were calculated from the dry organic matter content of each amendment to increase the amount of organic matter in each plot by up to 3% [40]. The organic amendments applied were: (i) 100% vegetable compost made from garden waste (COVG); (ii) 100% vegetable compost made from greenhouse horticultural crop residues (COHort); (iii) sewage sludge residues treated by mesophilic digestion, thermal dehydration at 70 °C and centrifugation (SS); (iv) 50:50 mixture of (i)+(iii) (COVG + SS); and (v) 50:50 mixture of (ii)+(iii) (COHort + SS). The main chemical characteristics of organic amendments and the studied soils are provided in Soria et al. (2022) [11]. In addition, three experimental plots without addition of organic amendments were used as Control (CON). Also, three areas close to the experimental plots, but undisturbed by mining activities, were also selected as replicates of “natural” quality reference soils (NAT) [41, 42].

Two weeks after application of the treatments, two native species from the area were planted in each plot (including the Control plots): 40 *Macrochloa tenacissima* L. Kunth and 10 plants of *Olea europaea* L. var. sylvestris Brot. with a 1 m planting frame. Due to the harsh environmental conditions (high temperatures and drought) of the area [34], an initial drip irrigation of 3 L per plant was applied after planting to ensure plant survival. Subsequently, 1 L per plant was provided every two weeks until the end of August, totaling five irrigation events. After this period, the plants relied solely on rainwater. More information on plant growth and survival can be found in Soria et al., 2021 [43].

After six months (17-12-2018) of the application of the organic amendments, the soil was sampled from the mixture of 10 subsamples randomly collected in each plot to a depth of 10 cm. A total of 21 samples were collected consisting of 5 treatments (3 replicates per treatment), 3 Controls and 3 Natural soils. Soil samples were stored in isothermal bags with ice and transported to the laboratory. Once there, a subsample was air dried, homogenized and sieved with a 2 mm sieve to determine soil physical and chemical properties. Another subsample was also homogenized, fresh sieved at 2 mm and one fraction stored at 4 °C for biochemical assays and another at -20 °C for DNA extraction and high-throughput sequencing.

### Soil chemical, physical and biochemical assays

The electrical conductivity (EC) and pH were measured in 1:2.5 (w/v) aqueous soil extracts using a digital conductivity meter and a pH meter (LAQUA, HORIBA, Tokyo, Japan), respectively. Soil content of total organic carbon (TOC) was determined by the method of Mingorance et al. (2007) [44] (colorimetric modification of Walkey and Black (1934) [45]. Total nitrogen (TN) was measured by total combustion with an Elemental Analyzer (Elementar Rapid N; Elementar Analysensysteme GmbH, Hanau, Germany) at the analytical services of University of Almeria. Assimilable phosphorus (AP) was determined using the procedure outlined by Olsen et al. (1954) [46]. Soil water holding capacity was assessed at pF values of -1500 and − 33 kPa using the Richards membrane method [47].

Soil basal respiration was measured by weighing 20 g of fresh mass into 125 ml vessels, with the water content adjusted to 50% of the water holding capacity. The vessels were then tightly sealed and incubated at 28 °C for 31 days. During this period, CO₂ production was periodically monitored by analyzing the gas composition in the vessels using an infrared gas analyzer (CheckMate 4; PBI Dansensor, Ringsted, Denmark). Urease activity (E.C. 3.5.1.5) was analyzed through the method developed by Kandeler & Gerber (1998) [48] and alkaline phosphatase (EC 3.1.3.1) following the Tabatabai & Bremmer (1969) method [49]. Soil bacteria acid methyl esters were extracted from 3 g of soil according to Schutter and Dick (2000) method [50], analyzed and quantified with a gas chromatograph (TRACE Ultra CG, Thermo Scientific, Waltham, Massachusetts, US), and was used to assess the abundance of bacteria.

### DNA extraction, metagenomic library preparation and sequencing

Full protocol details are available in the original previous paper [37] where the sequencing files were generated and submitted to the SRA. DNA extraction was performed from the 21 soil samples with the DNeasy PowerSoil kit (Qiagen, Hilden, Germany), using 0.3 g of soil and quantification was done with an ND-2000 Nanodrop spectrophotometer (Thermo Fisher Scientific, USA). Sequencing was conducted at the Integrated Microbiome Resource (IMR; Dalhousie University) using the Illumina Nextera DNA Flex kit with Nextera DNA CD indices, followed by sequencing on an Illumina NextSeq 550. The raw metagenomic sequences reanalyzed in this study are available at the ENA under accession number PRJEB47869.

Regarding metagenomic taxonomical and functional data, an initial bulk analysis of data was reported on in our previous study [37], outlining the broad general characteristics of the carbon cycle and overall taxonomic profiles of the samples. Briefly, an average depth of 5 million PE reads/sample were subjected to quality control and then were assigned function and taxonomy using our in-house developed SOP (https://github.com/LangilleLab/microbiome_helper/wiki/Metagenomics-Standard-Operating-Procedure-v3), which incorporates MMseqs2 and Kraken2. For this study, we utilized the already-generated normalized RPKM (Reads Per Kilobase per Million reads mapped) tables to complete our more in-depth analysis.

Firstly, to complete the metabolic mapping, the unstratified (function only) RPKMs values related to EC function numbers were summed across replicates to obtain an aggregate file with seven sample columns based on the different soil types (Natural, Control, COVG, COHort, SS, COVG + SS, COHort + SS). This table served as input for KEGGCharter ver.1.1.1 [(51] which serves as an automated way to annotate KEGG metabolic maps with functional potential (presence/absence in the Additional file 1 and 2) or differences (levels of RPKMs) between samples. Visual mappings were generated for KEGG pathway map00440 = Phosphonate and Phosphinate Metabolism (P) and pathway map00910 = Nitrogen Metabolism (N). The P map contains a total of 31 EC numbers of interest and the N map contains 39, listings of which are available through the KEGG REST API (https://rest.kegg.jp/link/enzyme/pathway: map00440; https://rest.kegg.jp/link/enzyme/pathway: map00910). RPKM values were also plotted with ggplot2 ver.3.5.1 (https://ggplot2.tidyverse.org/) in R ver.4.3.3 (https://www.r-project.org/) for direct comparison of the relative abundances.

Secondly, for the taxa-function visualizations, the stratified (function + taxa) RPKM tables were utilized as input for our in-house JarrVis tool (https://github.com/dhwanidesai/JarrVis), according to the instructions on the tools site and our above-mentioned MGS SOPv3. Simple scripting was used to pull out only those lines containing data for the EC numbers included in KEGG map00440 (P) or map00910 (N). Replicates were not summed here as the JarrVis tool automatically aggregates samples for visualization according to the provided metadata treatment designations. To improve readability of the Sankey plots, “noise” (very low RPKM contributions) was optionally filtered out using a set threshold RPKM value (indicated in the figure legends when applied). Numbers reported in the text are, however, the unfiltered values. The taxonomic contributions were verified using our in-house tool CoverageChecker v0.0.1-beta (https://github.com/R-Wright-1/genome_coverage_checker/). Details are available on the GitHub site (and a forthcoming manuscript), but briefly it verifies the Kraken2 results by mapping the raw reads back to reference genomes of the species assigned by Kraken2. For the purposes of this study, the CoverageChecker results were aggregated at the genus level (to match tables and figures) and the genera were considered potentially spurious taxonomic labels if at least one of the species within that genus did not have at least 1% genome coverage or, for rare members of the community, at least 100 reads found by Kraken2 with at least 50% confirmed mapping results by CoverageChecker within any one treatment. Full results are available in Additional files 3 and 4.

The samples were grouped according to the soil treatment factor (restored soils (COVG, COHort, SS, COVG + SS and COHort), mined soils without amendments (CON) and unmined natural reference soils (NAT)). Significant differences in physical, chemical and biochemical properties, potential functions in each biogeochemical cycle (N and P) and taxa involved in the main potential functions were evaluated using univariate and multivariate permutational analysis of variance (PERMANOVA, respectively, with 999 simulations, p-value < 0.05) [52] for the aforementioned factors. The Euclidean distance similarity matrix was used to analyze the physical, chemical and biochemical properties. The Bray-Curtis distance similarity matrix was used for potential functions and taxa. Pairwise comparisons were made using a multivariate analog of the t-t-test, and the probability levels were found by permutation [53], using the Monte-Carlo test when the number of free permutations was less than 100.

To determine if the structure of the potential functions (RPKM) was affected by the treatments, a principal coordinate analysis (PCoA) was developed. In PCoA graphs, the distance between points is proportional to the similarity of the functional profiles in each treatment and sample [54]. Non-metric Multidimensional Scaling analysis (NMDS) was performed to determine the structure of the bacterial communities at the genus level associated with the different metabolic pathways studied. Pearson correlations patterns (p-value < 0.05) between the potential dominant functions and soil properties for different treatments were carried out to explore the relationships between the effects of the treatments on soil properties and the dominant functions in each biogeochemical cycle.

The statistical package PRIMER + PERMANOVA (PRIMER-E Ltd., Plymouth Marine Laboratory, UK) for Windows was used for PERMANOVA, PCoA and NMDS routines. The R package ‘ggplot2’ (R Core Team) was used to create the bar charts, correlation charts, heatmaps and Sankey plots.

## Results

### Soil chemical, physical and biochemical properties

**Fig. 1:**
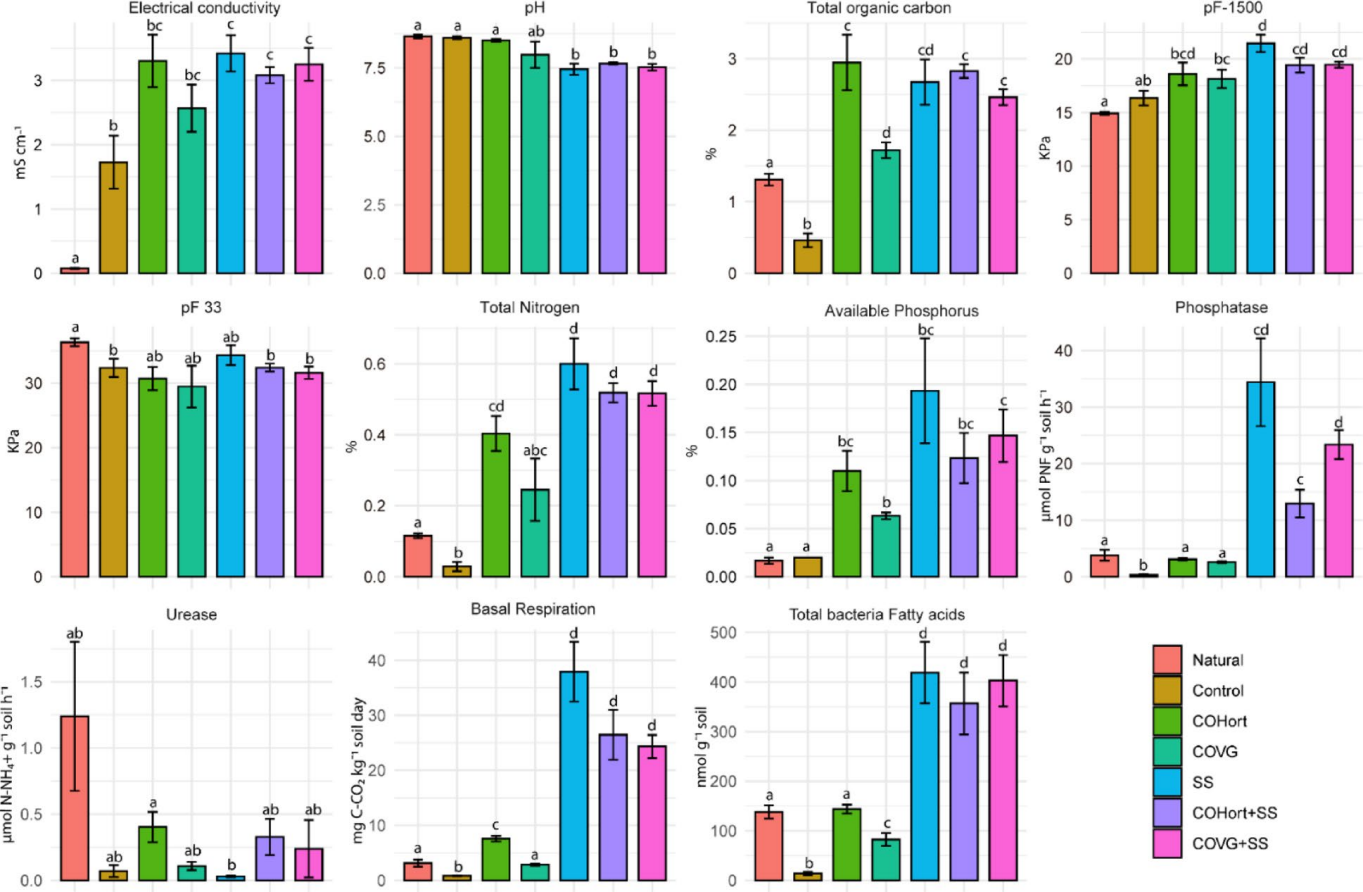
Main physical, chemical and biochemical properties of soils restored with organic amendments, controls, and natural reference soils (mean ± standard error) soils. Different lowercase letters indicate significant differences (*p* < 0.05) among the treatments according to the PERMANOVA pairwise test comparisons. Natural: non-mined reference soils; Control: unamended control soils; COVG: vegetable compost from garden waste; COHort: vegetable compost from greenhouse crop residues; SS: wastewater treatment sludges from anaerobic mesophilic digestion, dehydrated by centrifuge and heat dried at 70 °C; COVG + SS and COHort + SS: mixtures of different vegetal composts and sludge compost

In general, organic amendments significantly changed the chemical, physical and biochemical properties in treated soils compared with Control and Natural soils (Additional file 1-Table S1). As expected, due to the characteristics of organic amendments, EC clearly increased and pH decreased compared to unamended soils (Control and Natural), especially in soils with SS and their mixtures with vegetable compost. In addition, amended soils showed a significantly higher nutrient content. These increases occurred mainly in Total Organic Carbon (TOC), where soils treated with COHort and SS and mixtures showed mean values of 2.48% and 2.19% higher than Control soils, and 1.64% and 1.34% higher than Natural soils, respectively. Among the treated soils, the lowest values were presented for COVG, which increased TOC levels by 1.72% compared to Control and similar to Natural. For total nitrogen (TN), SS and their mixtures showed the higher values, followed by COHort and COVG and Natural, while Control presented the lowest values. Available phosphorus (AP) followed the same pattern for the treated soils, however Natural and Control in this case presented similar values. Moreover, all soils treated with organic amendments showed significantly higher water retention capacity at − 1500 kPa compared with unamended soils. The application of organic amendments was also effective in stimulating soil microbiota and growth, showing significantly higher basal respiration in treated plots compared with Control. Specifically, soil treated with SS and their mixtures, followed by COHort, were higher while COVG presented a significantly lower basal respiration rate similar to Natural soils. Higher activity was also shown with the same trend for alkaline phosphatase activity, with SS treated soils being the most active, while both vegetable composts were the least, but still all superior to all untreated soils. Despite the fact that there were no significant differences in urease activity, the Natural soils trended towards comparatively higher activity than the treated soils. In addition, the abundance of soil bacteria determined from fatty acid analysis showed that soils treated with SS and mixtures had a significantly higher bacterial biomass, following by COVG and Natural soils. COHort showed the lowest values among the treated soils, and Control soils presented the significant lower values (Fig. 1).

## Potential N functions in the soils

**Fig. 2:**
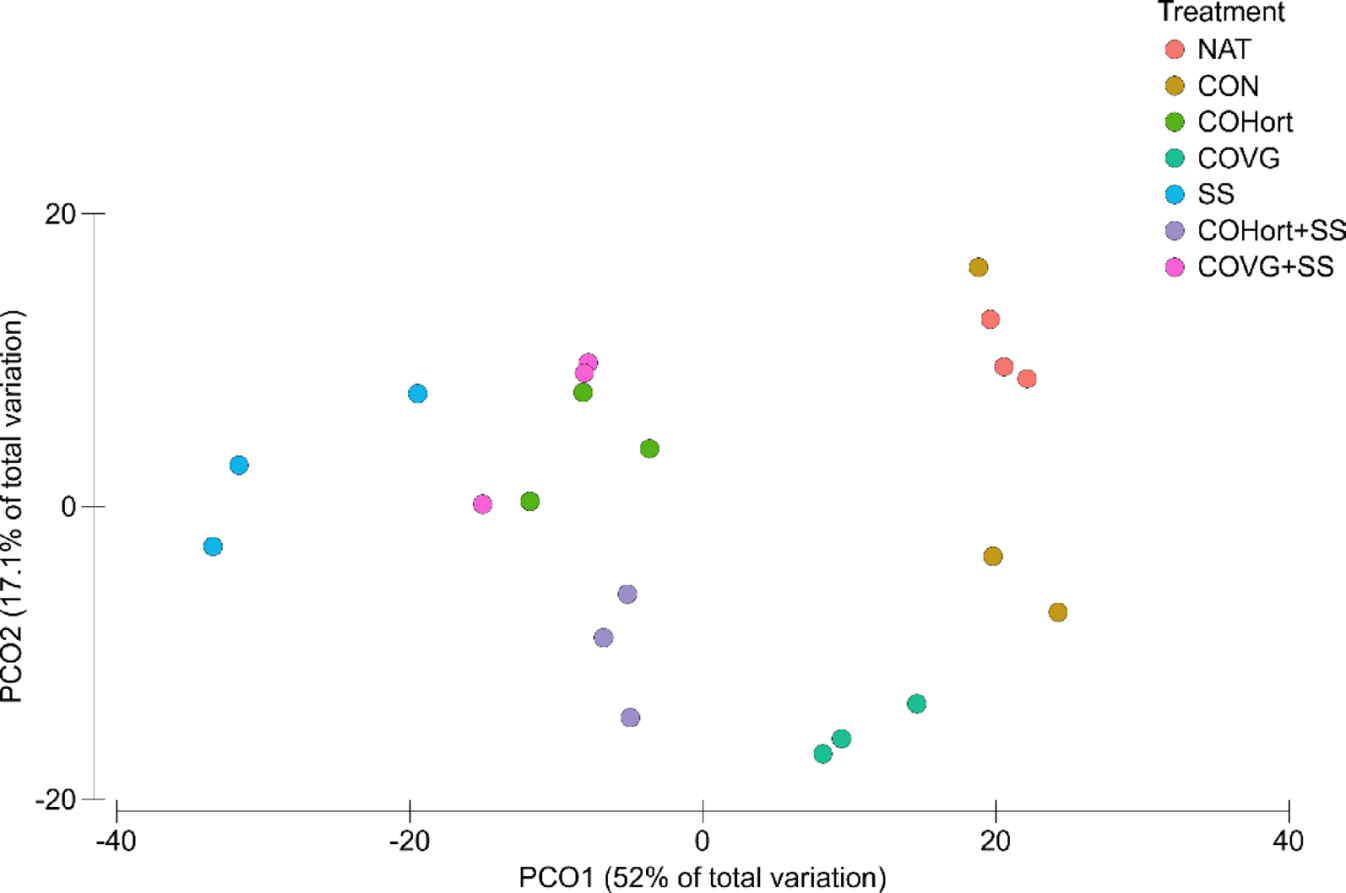
Ordination by principal coordinate analysis (PCoA) based on Bray-Curtis distance showing the effects of different treatments on potential bacterial functions in the N cycle, compared to untreated (Control) and natural reference soils (Natural). COVG: vegetable compost from garden waste; COHort: vegetable compost from greenhouse crop residues; SS: wastewater treatment sludges from anaerobic mesophilic digestion, dehydrated by centrifuge and heat dried at 70 °C; COVG + SS and COHort + SS: mixtures of different vegetal composts and sludge compost

From the MGS data and using the KEGG database, a total of 32 out of 39 potential functions in N metabolism were identified. Significant differences (*p* < 0.05) were observed between potential genes (based on RPKM contributions) in soils treated with organic amendments and unamended soils (Additional file 1-Table S2). PCoA analysis showed that organic amendment treatments altered the potential functional patterns (Fig. 2; Additional file 1-Table S3). The first two axes of the PCoA explained 69.1% of the variations in the potential functions by treatment (PCO1 = 52%; PCO2 = 17.1%). Samples from treated soils (COHort, SS, COHort + SS and COVG + SS) clustered separately form Control and Natural soils, located at opposite sides along PCO1, except for COVG treated soils with were an intermediate position among unamended and treated soils. Within the cluster formed by the samples of soils treated with organic amendments, a clear separation was observed between the treatment with sewage sludge (SS) and the other treatments, including their mixtures (COHort + SS and COVG + SS) and the compost derived from horticultural residues (COHort) (Fig. 2).

**Fig. 3:**
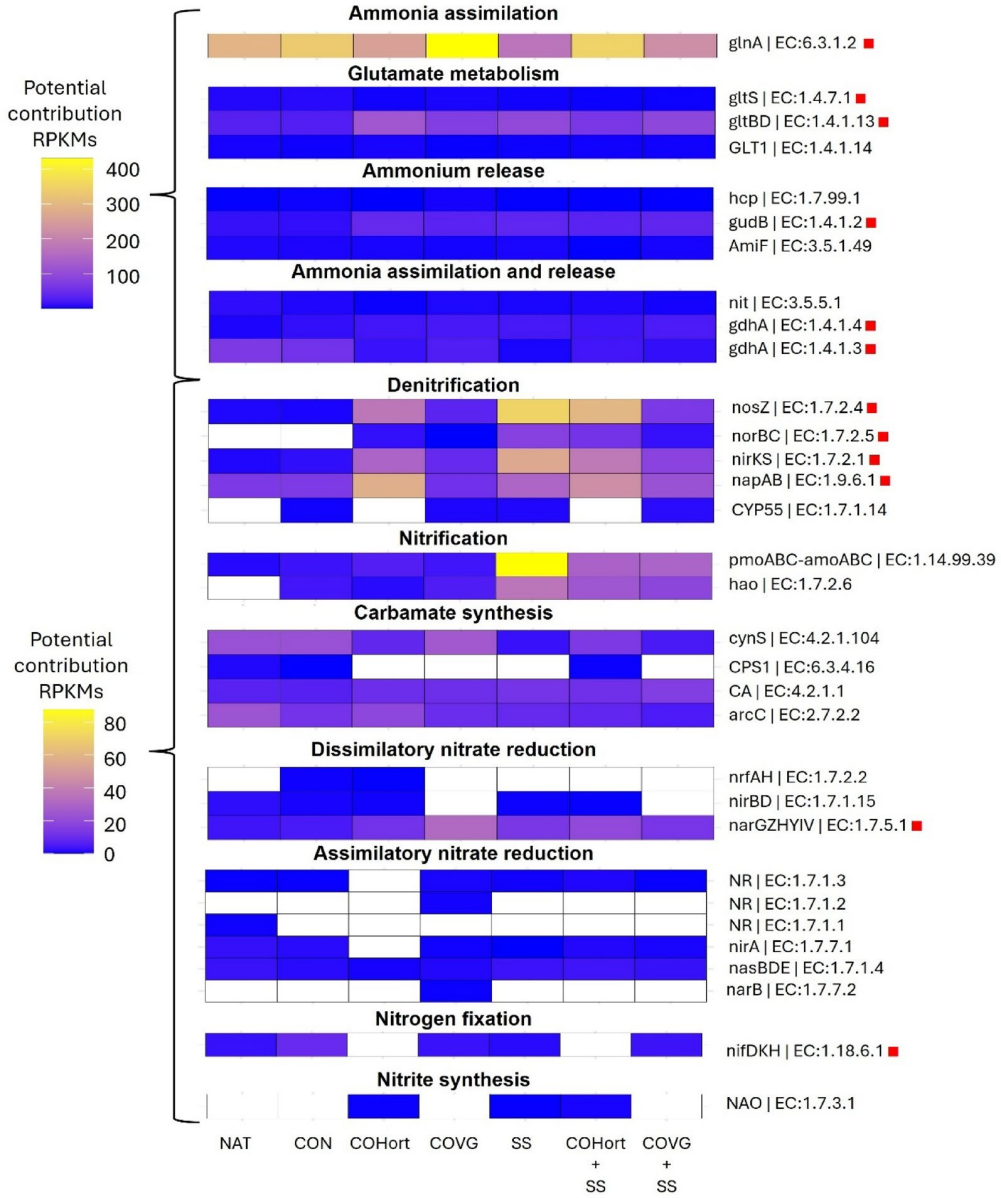
Heatmap of the contribution (average RPKMs) of potential functions associated with nitrogen metabolism. The potential functions were subdivided depending on the process in which they participate in nitrogen metabolism. The red squares (■) on the right side of the name of the potential functions indicate differences (*p* < 0.05) among the treatments according to the PERMANOVA pairwise comparisons test. Natural: non- mined reference soils; Control: unamended control soils; COVG: vegetable compost from garden waste; COHort: vegetable compost from greenhouse crop residues; SS: wastewater treatment station sludges from anaerobic mesophilic digestion, dehydrated by centrifuge and heat dried at 70 °C; COVG + SS and COHort + SS: mixtures of different vegetal compost and sludge compost

The main potential functions detected involved processes such as ammonium assimilation and glutamate metabolism (Fig. 3, S1). Additionally, potential functions associated with denitrification, nitrification, assimilatory and dissimilatory nitrate reduction, and N fixation were identified, but with lower contribution (Fig. 3). Soils treated with organic amendments showed significant high RPKM contributions (COHort + SS = 759 ± 99, COVG = 723 ± 37, SS = 687 ± 79, COHort = 647 ± 101, COVG + SS = 549 ± 162) compared to Control (541 ± 41) and Natural reference soils (520 ± 42) (Additional file 1-Table S2). The contribution of potential functions to the total observed RPKM was predominantly driven by *glnA* (glutamine synthetase; EC:6.3.1.2), which accounted for 47% of the total RPKMs and is associated with ammonium assimilation. Following this, *gltBD* (glutamate synthase (NADPH); EC:1.4.1.13) contributed 12%, a function involved in glutamate metabolism. Other significant contributors included *gudB* (glutamate dehydrogenase; EC:1.4.1.2), and *gdhA* (glutamate dehydrogenase (NAD(P)+); EC:1.4.1.3), with contributions of 5% and 4%, respectively, both related to ammonium release or assimilation. Additionally, functions such as *napAB* and *nosZ* (nitrate reductase; EC:1.9.6.1 and EC:1.7.2.4) accounted for 4% and are associated with the denitrification process. pmoABC-amoABC (ammonia monooxygenase; EC:1.14.99.39) also contributed 4% of the total RPKM and is involved in the nitrification process. The remaining proportion of RPKMs was distributed among 25 other potential functions, each contributing less than 4% (Fig. 3 and Additional File 1-Table S4).

Soils with organic amendments showed the highest contributions to glutamate metabolism, primarily driven by the potential transcription of *gltBD*. These soils also had a greater proportion of ammonium release and dissimilatory nitrate reduction gene content, with significantly higher potential contributions from the *gudB* and *narGZHYIV* genes. A similar trend was observed in the denitrification process, where amended soils, especially those containing sludge (SS) and its mixtures (COHort + SS, COVG + SS), showed significantly higher values compared to unamended soils, highlighting the potential of *nosZ*, *norBS*, *nirKS* and *napAB* genes in these treatments, with values two to three times higher than unamended soils. Regarding nitrification, SS-treated soils had the most significant contributions, followed by COHort + SS and COVG + SS, which showed intermediate contributions. On the contrary, soils without organic amendments (Control and Natural) showed the highest contributions in ammonium assimilation and release, particularly represented by *gdhA*. Additionally, Control soils showed comparatively higher contributions to nitrogen fixation, with significant proportions of the *nifDKH* genes (Fig. 3 Additional File 1-Table S5).

## Taxonomic contributions to potential N functions in the soils

A total of 41 phyla and 1175 taxa at the genus level were identified that contribute to 32 potential functions in N cycling (Additional file 1-Table S2). Most of the affiliated sequences (97%) were of bacterial origin (Fig. S2a). At the phylum level, *Pseudomonadota*,* Actinomycetota*,* Bacillota* and *Deinococcota* were more abundant in the amended and unamended plots (Fig. S2b). Comparing the different treated soils, the soils with SS had a higher abundance of *Pseudomonadota*, followed by COHort and the mixtures (COHort + SS and COVG + SS), while in COVG, *Actinomycetota* predominated. In unamended soils, Natural soils have a similar proportion of *Actinomycetota* and *Pseudomonadota* phyla, while in Control soils the *Actinomycetota* phylum predominated.

**Fig. 4:**
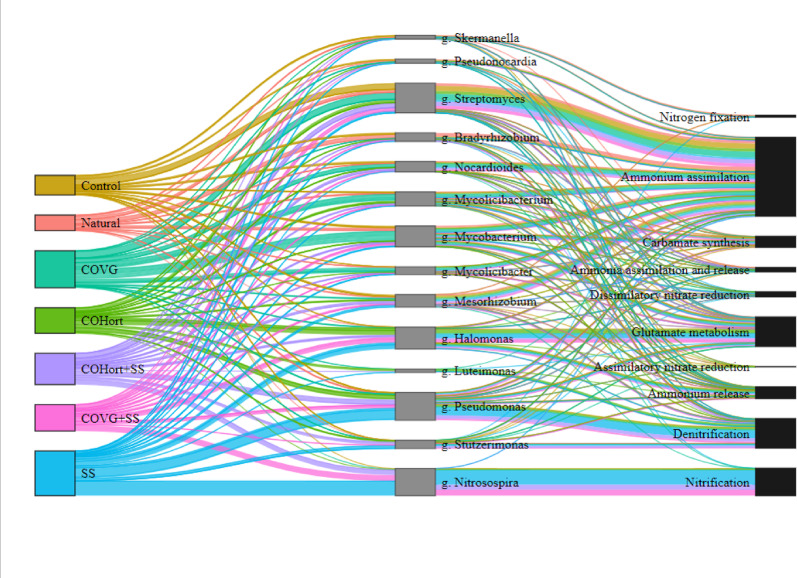
Stratified potential functions of N metabolism showing quantitative relationships between treatments (left-color-coded), top 5 genera per treatment (middle-gray) and associated metabolic processes (right-black). The lines joining both sides through the detected genera are proportional to the total sum of RPKM values in the samples and to the contributions of the potential functions of each genus. The thickness of the links represents the magnitude of the contribution. All genera listed here were confirmed by CoverageChecker. Natural: non- mined reference soils; Control: unamended control soils; COVG: vegetable compost from garden waste; COHort: vegetable compost from greenhouse crop residues; SS: wastewater treatment station sludges from anaerobic mesophilic digestion, dehydrated by centrifuge and heat dried at 70 °C; COVG + SS and COHort + SS: mixtures of different vegetal compost and sludge compost

At the community structure level, PERMANOVA analysis revealed significant differences in the taxonomic composition of microbial communities at the genus level between treatments (Additional File 1 – Table S6), aligning with the NMDS ordination of soil samples (Fig. S2c). *Streptomyces* was the most abundant genus, being predominant in Control soils, however it also had a high relative abundance in COVG-treated soils, together with others such as *Mycolicibacterium* and *Mycobacterium*. Soils amended with COHort showed a relative abundance of *Streptomyces* similar to that of Natural soils and mixtures (COHort + SS and COVG + SS), with values 50% lower than Controls. SS showed 80% less *Streptomyces* than Controls, instead *Nitrosospira*, *Pseudomonas* and *Halomonas* were the most abundant genera. Soils modified with a mixture of sludge amendments and the two plant composts often showed relative abundances of taxa similar to the original composts without the sludge (Fig. S2d). The five most abundant genera for each treatment were selected and depicted in a Sankey diagram, linking them to the metabolic processes to which they contributed (Fig. 4). Although potential functional redundancy was also observed, marked patterns where soils treated with organic amendments differed from untreated soils were evident (Fig. 4). Specifically, *Streptomyces*,* Mesorhizobium*,* Nocardioides and Mycobacterium* presented a greater potential contribution to the assimilation of ammonium, while *Halomonas*,* Mycobacterium and Pseudomonas* did so in the metabolism of glutamate and ammonium release. The denitrification process was mainly mediated by *Pseudomonas*, *Stutzerimonas and Luteimonas* while nitrification was almost exclusively by *Nitrosospira.* Less abundant genera such as *Skermanella* contributed to nitrogen fixation, as *Mycolicibacter* did to dissimilatory nitrate reduction.

### Interrelationships between soil properties and nitrogen-related functional genes

**Fig. 5:**
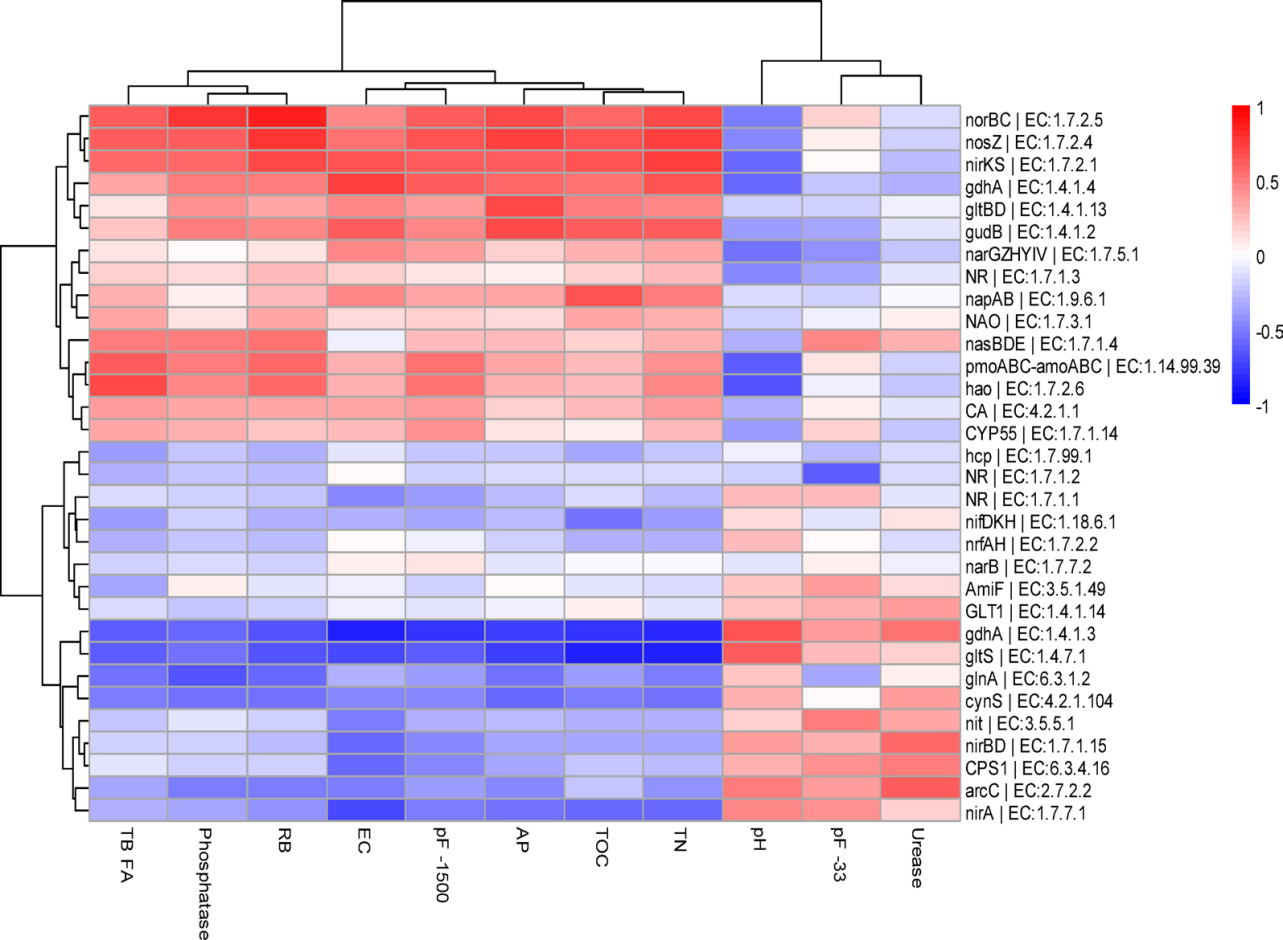
Pearson correlations (*p* < 0.05) between soil physical, chemical and biochemical soil properties and potential genes involved in N metabolic pathways in different soils treated with organic amendments and untreated. EC: electrical conductivity; TOC: total organic carbon; TN: total nitrogen; TB FA: Total bacteria fatty acids; Phosphatase: alkaline phosphatase enzyme activity; RB: Soil basal respiration; pF-1500 and pF-33: water holding capacity at 1500 and 33 kPa; Urease: urease enzyme activity; AP: available phosphorus.

Potential genes involved in denitrification processes, such as *norBC*, *norZ* and *nirKS* showed strong positive significant correlations with EC, TOC, TN pF-1500, RB and TBFAm, and negative with pH. The same patterns of correlations were observed for *gdhA* implicated in ammonium assimilation/release and *gudB* in ammonium release except for TBFA. *pmABC-amoABC* and *hao*, involved in nitrification processes, had a significant negative correlation with pH, and positive ones with pF-1500, TN, RB and TBFA. There were also genes such as *nirA* (assimilatory nitrate reduction), *gltS* (glutamate metabolism) and *gdhA* (ammonium assimilation/release) that showed the opposite correlation pattern, with significant positive correlations with pH and urease activity, but negative correlations with the rest of the variables (Fig. 5 Additional File 1 – Table S7).

## Potential P functions in the soils

**Fig. 6:**
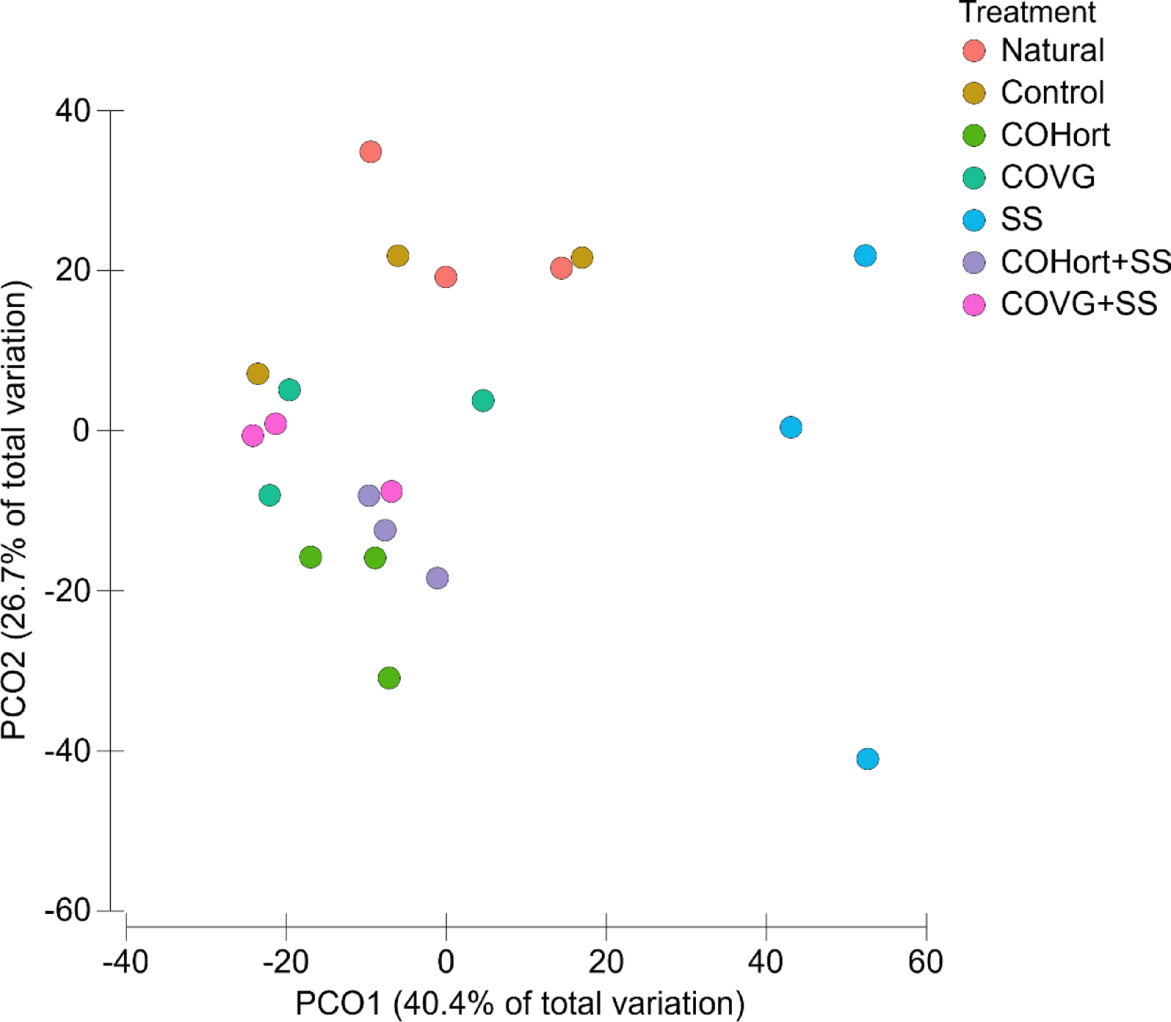
Ordination by principal coordinate analysis (PCoA) based on Bray-Curtis distance showing the effects of different treatments on potential bacterial functions in the P turnover (phosphinate and phophonate pathways), compared to untreated (Control) and natural reference soils (Natural). COVG: vegetable compost from garden waste; COHort: vegetable compost from greenhouse crop residues; SS: wastewater treatment sludges from anaerobic mesophilic digestion, dehydrated by centrifuge and heat dried at 70 °C; COVG + SS and COHort + SS: mixtures of different vegetal compost and sludge compost

A total of 17 out of 31 potential KEGG functions in P metabolism were detected in the samples. The results of PERMANOVA test showed significant differences among treatments (Additional file 2-Table S1). The PCoA analysis (total explained variance of 67.1%; PCO1 = 40.4% and PCO2 = 26.7%) clearly separated soils treated with sewage sludge (SS) from other treated and untreated soils throughout PCO1, while PCO2 grouped the Natural soils and Controls with positive values on the axis, fairly well separated from the rest of the treatments (COVG, COHort, COHort + SS and COVG + SS) (Fig. 6, Additional file 2-Table S2).

**Fig. 7:**
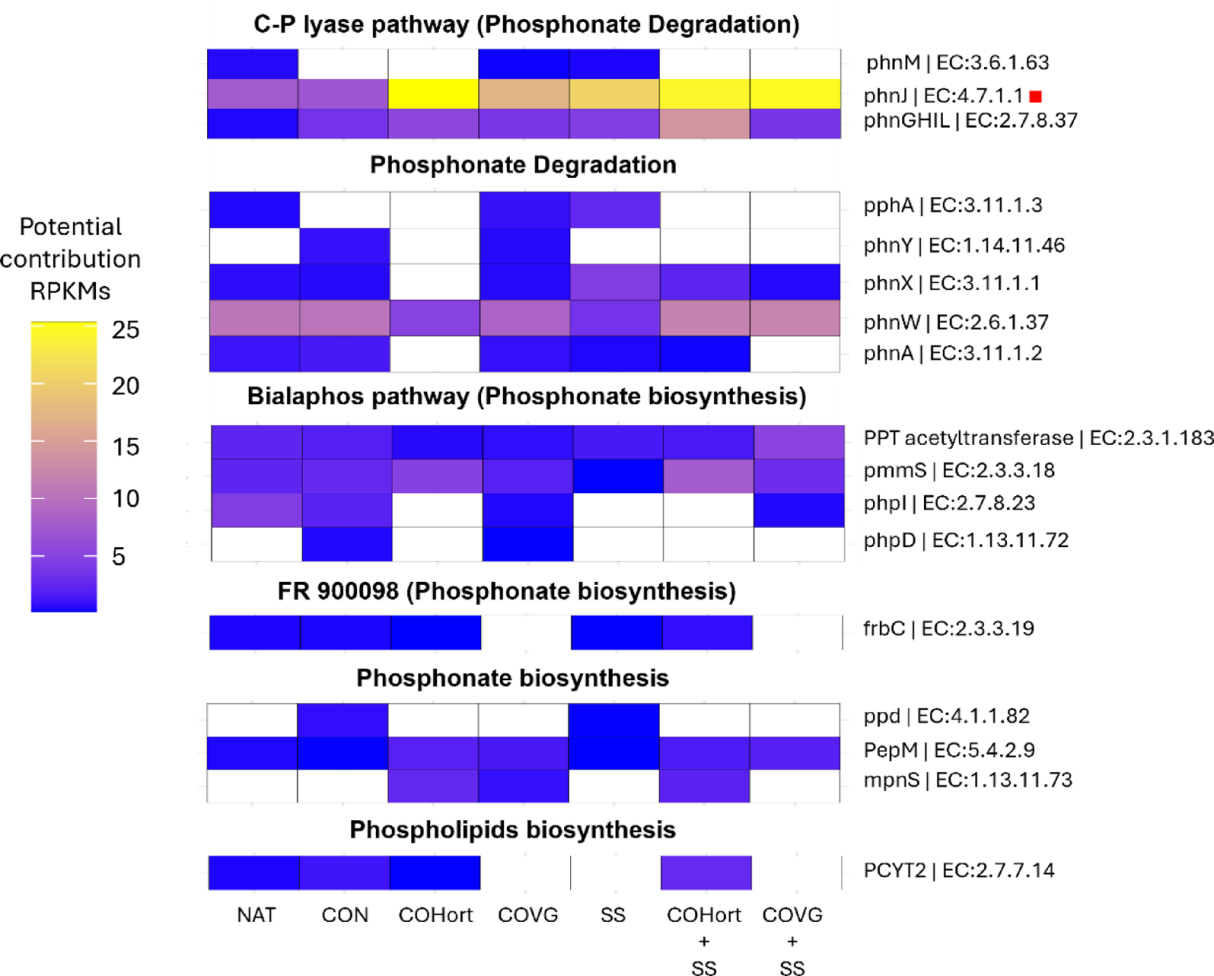
Heatmap of the contributions (average RPKMs) of potential functions associated with phosphonate and phosphinate metabolism. The potential functions were subdivided depending on the process in which they participate in phosphonate and phosphinate metabolism. The red square (■) on the right side of the name of the potential functions indicate significant differences (*p* < 0.05) among the treatments according to the PERMANOVA pairwise comparisons test. Natural: non-mined reference soils; Control: unamended control soils; COVG: vegetable compost from garden waste; COHort: vegetable compost from greenhouse crop residues; SS: wastewater treatment station sludges from anaerobic mesophilic digestion, dehydrated by centrifuges and heat dried at 70 °C; COVG + SS and COHort + SS: mixtures of different vegetal composts and sludge compost

The main potential functions detected were involved in the processes of phosphonate degradation, with the greatest potential contribution being the C-P-lyase pathway, although other metabolic pathways with a lesser contribution that lead to phosphonate degradation were also detected (Fig. S3). In addition, other potential functions associated with the biosynthesis of phosphonates through the metabolic pathway of bialaphos and other alternative pathways were identified, as well as the biosynthesis of phospholipids (Fig.S3). Although in general the potential contributions of the functional genes detected were very low, soils treated with organic amendments showed comparatively higher RPKM contributions (COHort + SS = 69 ± 22, COVG + SS = 52 ± 32, COHort = 46 ± 11, COVG = 38 ± 1, SS = 38 ± 16), compared to Control 33 ± 6 and Natural reference soils 31 ± 6 (Additional file 2-Table S1). The contribution of potential functions to the total observed RPKMs was predominantly driven by the *phnJ* gene (alpha-D-ribose 1-methylphosphonate 5-phosphate C-P-lyase; EC:4.7.1.1) and *phnGHIL* (alpha-D-ribose 1-methylphosphonate 5-triphosphate synthase, EC:12.7.8.37), which accounted for 41% and 12% of the total RPKMs respectively and were associated with phosphonate degradation through the breakdown of C-P bonds. Following this, the *phnW* gene (2-aminoethylphosphonate-pyruvate transaminase, EC: 2.6.1.37) contributed 20%, a function also involved in phosphonate degradation. Meanwhile, functions involved in phosphonate biosynthesis, specifically in the bialaphos pathway, such as *pmmS* (2-phosphinomethylmalate synthase, EC: 2.3.3.18) and *PPT* (phosphinothricin acetyltransferase, EC: 2.3.1.183) contributed 7% and 4% respectively. The remaining proportion was distributed among 12 other potential functions, each contributing less than 3% (Fig. 7 and Additional File 1-Table S3).

Soils treated with organic amendments showed a greater contribution to phosphonate degradation (C-P lyase), primarily driven by the *phnJ* gene, which exhibited a significantly higher contribution compared to unamended soils (Control and Natural). Similarly, although *phnGHIL* did not show significant differences, it displayed a trend of higher contribution in amended soils compared to unamended soils. The *phnW* gene showed higher contributions in unamended soils, COVG and mixtures. On the other hand, phosphonate and phospholipid biosynthesis had contributions below 10 RPKMs with the *pmmS* and *PPT* genes having the greatest potential contribution to this pathway (Fig. 7 and Additional File 2-Table S4). The treated soils (especially COHort + SS) showed a positive impact on the degradation and biosynthesis of phosphonates and phospholipids. COHort led the biosynthesis of phosphonates, while COVG + SS and COVG showed variable contributions depending on the metabolic process, with intermediate values in degradation and biosynthesis. SS had an outstanding contribution in degradation, but its participation in biosynthesis was lower compared to other treatments. In contrast, Natural and Control soils presented more variable contributions depending on the process analyzed, being more notable in the degradation of phosphonates (Fig. 7).

### Taxonomic contributions to potential P functions in the soils

A total of 13 phyla and 299 genus-level taxa were identified that contribute to the 17 potential functions in the P turnover, and more specifically to the metabolism of phosphonates and phosphinates (Additional file 2-Table S1). As for N cycling, most of the identified sequences (98%) were bacterial in origin (Fig. S4a). Overall, the most abundant bacterial phyla in all soils were *Pseudomonadota* and *Actinomycetota* (Fig. S4b). Interestingly, Natural soils showed a higher presence of *Deinococcota*, which also presented a higher relative abundance in soil modified with COVG compared to the rest of the treated soils, while *Bacteroidota* and *Myxococcota* were detected in COVG but absent in the rest of the treated and untreated soils.

PERMANOVA tests showed significant differences in the structure of the bacterial communities at the genus level (Additional file 2- Table S5), however the high variability within groups did not allow the NMDS analysis to clearly reflect the similarities between the samples of each treatment (Fig. S4c). *Streptomyces* was the most abundant genus, exhibiting the highest contribution in Natural soils, which was approximately 7 times greater than in Control and fluctuated across amended treatments, with the exception of COVG in which this genus was not found. *Sinorhizobium* predominated in soils with COVG, contributing approximately 13 times more than in other amended soils. Notably, this genus was absent in soils without amendments. In COHort, *Rhizobium* was the genus with the highest relative abundance, similar to its mixture (COHort + SS), being almost 7 times more abundant than in COVG and Natural soils and 2 times more abundant than the other treatments (Control, SS and COHort + SS). In these same soils treated with horticultural amendments (COHort) genera such as *Allorhizobium* showed abundances approximately 16 times higher than in SS and it was not detected in the other treatments. Similarly, *Bifidobacterium* had an abundance approximately 5 times higher than in COHort + SS and was absent in the other treatments. The soils treated with the COVG + SS mixture showed the predominance of *Bosea*, with a relative abundance approximately 10 times higher than COVG and unamended soils. Other genera present in this mixture were *Cohaesibacter*,* Pseudomonas*,* Streptomyces* and *Mesorhizobium.* For their part, the soils treated with the COHort + SS mixture showed a relative abundance of *Rhizobium* similar to COHort, and other abundant genera in this soil were *Pseudorhizobium* and *Devosia.* In Natural soils the most abundant genus was *Streptomyces*, followed by *Paraburkholderia* and *Bradyrhizobium*, while in Control soils it was *Mesorhizobium*, followed by *Rhizobium* and *Burkholderia.*

**Fig. 8:**
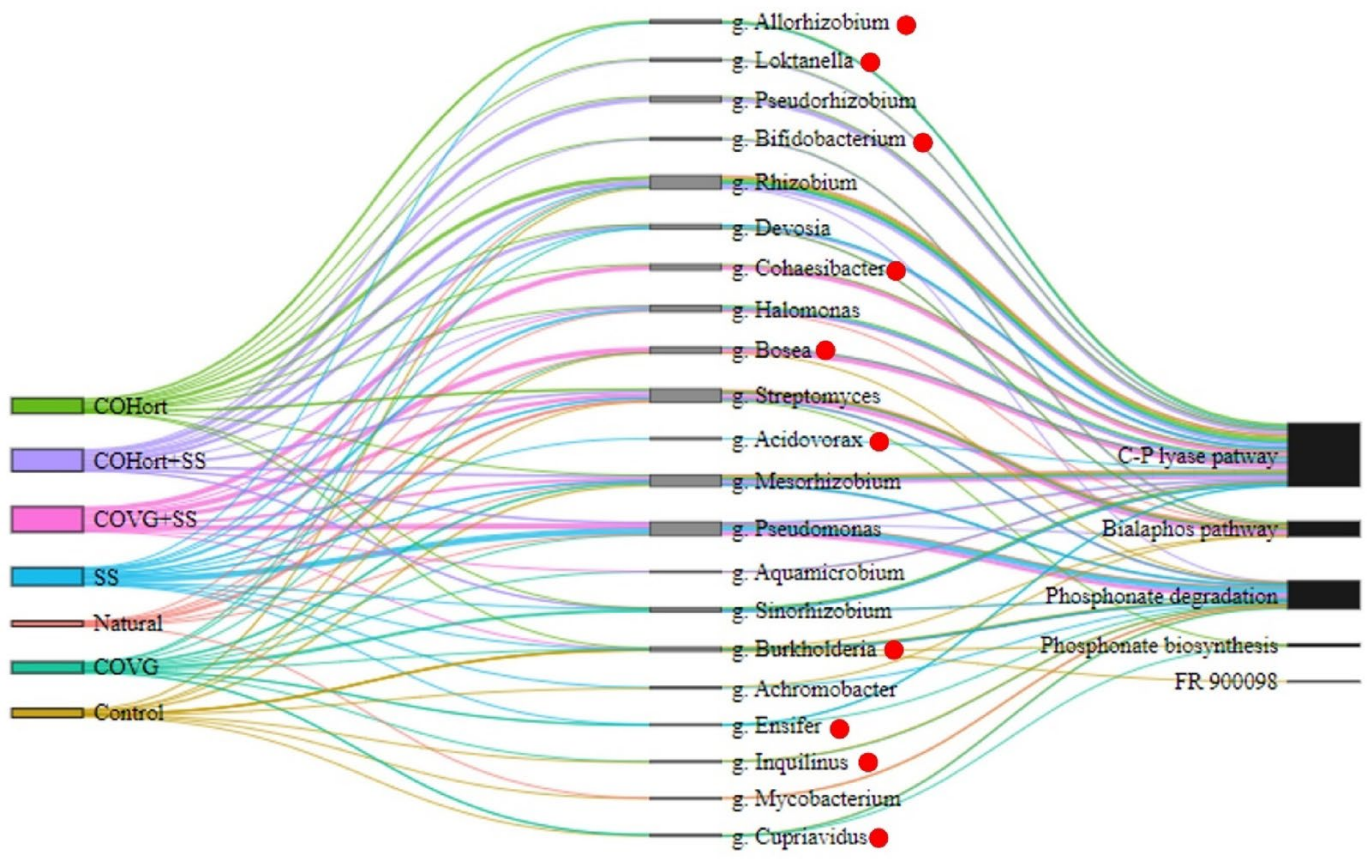
Stratified potential functions of phosphonate and phosphinate metabolism showing quantitative relationships between treatments (left-color-coded), top 5 genera per treatment (middle-gray) and associated metabolic processes (right-black). The lines joining both sides through the detected genera are proportional to the total sum of RPKM values in the samples and to the contributions of the potential functions of each genus. The thickness of the links represents the magnitude of the contribution. A portion of the genera (indicated by red dots) may be spurious taxonomic assignments, based upon CoverageChecker. not being able to confirm the Kraken2 results and so should be treated with caution. Control: unamended control soils; COVG: vegetable compost from garden waste; COHort: vegetable compost from greenhouse crop residues; SS: wastewater treatment station sludges from anaerobic mesophilic digestion, dehydrated by centrifuges and heat dried at 70 °C; COVG + SS and COHort + SS: mixtures of different vegetal composts and sludge compost

The five most abundant genera for each treatment were selected and depicted in a Sankey diagram, highlighting their potential contribution to metabolic phosphorus processes (Fig. 8). *Pseudomonas* mainly contributed to phosphonates degradation, followed by genera such as *Burkholderia*,* Mesorhizobium*, and *Streptomyces*, which contributed to a lesser extent. Regarding phosphonate degradation through the C-P lyase pathway *Rhizobium* was the genus with the highest contribution, followed by *Mesorhizobium*,* Bosea*,* Halomonas*,* Cohaesibacter* and *Pseudorhizobium.* Additionally, *Streptomyces* played a significant role in the synthesis of phosphonate and bialaphos pathway with lesser contributions from *Burkholderia* and *Rhizobium.* Furthermore, *Burkholderia* also made a small contribution to the biosynthesis of phosphonates and FR 900,098. Unlike the major taxa contributing to the N cycle in Fig. 4 which were all confirmed, some of these P cycle taxa may be misidentified as they did not pass the CoverageChecker threshold (indicated by red dots; see Methods). The major contributors were confirmed (such as *Rhizobium*, *Streptomyces*, and *Pseudomonas*), however smaller contributors such as *Bifidobacterium* and *Burkholderia* did not have enough reads mapped to their reference genomes by CoverageChecker to pass our cutoff level.

### Interrelationships between soil properties and phosphorus-related functional genes

**Fig. 9:**
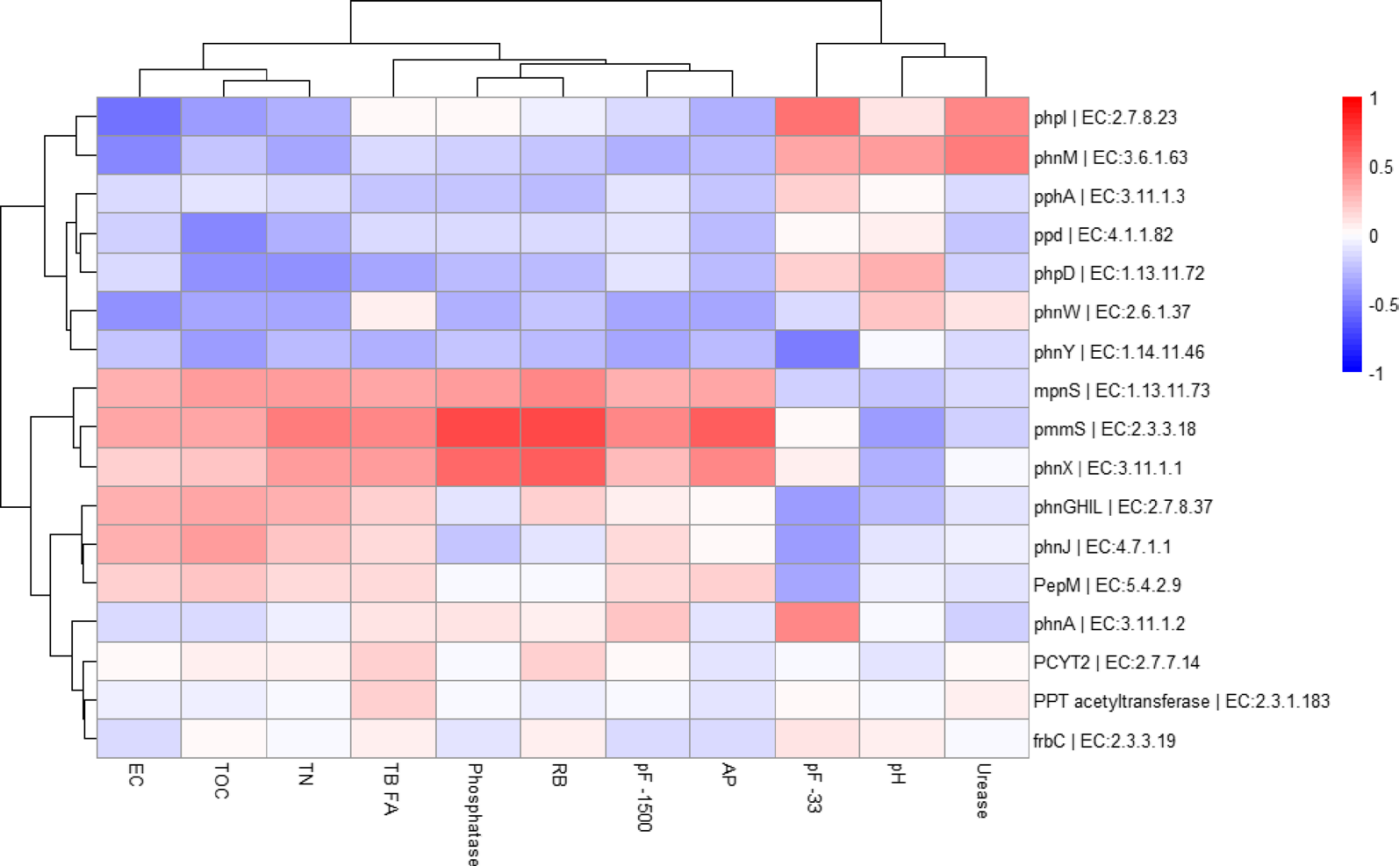
Pearson correlations (*p* < 0.05) between soil physical, chemical and biochemical soil properties and potential genes involved in P metabolic pathways in different soil treated with organic amendments and untreated. EC: electrical conductivity; TOC: total organic carbon; TN: total nitrogen; TB FA: Total bacteria fatty acids; Phosphatase: alkaline phosphatase enzyme activity; RB: Soil basal respiration; pF-1500 and pF-33: water holding capacity at 1500 and 33 kPa; Urease: urease enzyme activity; AP: available phosphorus

The *pmmS* gene involved in the biosynthesis of phosphonates through the bialaphos metabolic pathway showed the highest number of significant correlations with soil properties, specifically with available phosphorus (AP), alkaline phosphatase activity, basal respiration (BR), water retention capacity (pF-1500 kPa), total nitrogen content and (TN) and bacteria fatty acids (Fig. 9). In the same pathway, *phpI* showed significant positive correlations with urease activity and pF-33 kPa and negative with electrical conductivity (EC). On the other hand, *phnX*, with functions related to the phosphonate degradation process, showed significant positive correlations with AP, phosphatase activity and RB. Total organic carbon showed weaker and non-significant positive correlations with *pmmS*, *phnX* and *mpnS*, while it correlated negatively with *phpD*, *phnW* and *phnY* (Fig. 9).

## Discussion

MGS enabled an in-depth analysis of the proliferation of microbial communities and their potential roles in nitrogen (N) and phosphorus (P; phosphonates and phosphinates metabolism specifically) turnover in soils restored with organic amendments in a limestone quarry in a semiarid climate. Our results have shown a short-term improvement in soil quality in terms of increases of organic matter, and consequently of nutrients, as well as microbial activity after the application of amendments compared to untreated soils. Additionally, the application of organic amendments had a clear effect on the structure, composition and potential functionality of the microbial community, with higher representation of bacterial genera involved in N and P metabolism and establishing different taxa-function relationships depending on the type of organic amendments used.

Previous studies have reported that organic amendments regulated both the structure of microbial communities and their functional contributions to soil processes [55, 56], but its functional metabolic response varies depending on the nature and chemical composition of the organic amendments used [57, 58]. Our results showed different functional profiles in the different soil treated with organic amendments (Figs. 2 and 6), indicating metabolic variations linked to the adaptation of the microbiota to the new edaphic conditions driven by the amendments [59]. In our study, the application of organic amendments significantly increased TOC, TN, and AP content, water holding capacity, promoted bacterial biomass growth and stimulated microbiological activity compared to untreated soils. These results are consistent with previous studies that have reported similar effects after organic amendments application in semiarid ecosystem [60–63]. Concretely, soils treated with sludge (SS) and their mixtures with plant compost (COVG + SS and COHort + SS) showed the greatest changes in soil properties, providing higher nutrient content (TOC, TN and AP), lowering the pH and showing significantly higher bacterial growth, basal respiration and enzyme activity compared with soil restored with plant compost and Natural soils (Fig. 1). Given that amendments affect soil physical and chemical properties in different ways [64] and, in turn, soil properties such as pH, EC, nutrients content, water availability and microbial biomass have been widely described in the literature as key drivers of the structure and functionality of the soil microbiota, among others [65–70], the results obtained in the present study corroborate that microorganism-mediated changes in the soil functional profiles in the restored experimental plots were consistent with complex interactions among physical, chemical and biological soils properties. Organic amendments provide an input of organic matter to the soil, which is key for the development of soil functions [71, 72] and the maintenance of its quality and fertility [73]. Moreover, it is known that organic amendments contribute to increasing the growth of bacterial biomass in the soil, as they provide new communities of bacteria specific to each amendment [74, 75]. Soil microbial communities are fundamental to land restoration, as they play an essential role in the nutrient cycle and in plant establishment [76, 77]. The inputs of organic amendments in the experimental degraded quarry soils promoted changes in the microbiota associated with the different metabolic cycles studied. Although soils can harbour diverse types of microbial structures (fungi, archaea, bacteria, protists, etc.), and MGS is unbiased in profiling all the DNA in samples, the present study focused on soil bacteria as they were the majority group identified with involvement in the nitrogen and phosphorus metabolic pathways studied, with more than 95% of bacterial taxa identified as opposed to other type of microorganisms (Fig. S2a and S4a). In addition, soil bacteria are key transformers of nutrients in the soil and play an essential role in biogeochemical cycling [78, 79].

Interestingly, the majority of microbial taxa involved in nitrogen and phosphorus metabolisms at the phylum level were similar in both treated and untreated soils, suggesting that dominant groups such as *Pseudomonadota* and *Actinomycetota* function as generalist species with diverse ecological roles [80–82]. Additionally, these phyla are considered part of a typical oligotrophic microbial group, characterized by high nutrient affinity and strong adaptability to environments with limited nutrient availability [83]. However, *Pseudomonadota* had a main contribution in phosphonates metabolisms compared with other phyla, and have been previously highlighted as important phosphonate-consuming taxa [84]. On the other hand, in N metabolism its most significant contribution was associated with soil amended with sludge (SS). Malik et al. (2024) have reported the presence of *Pseudomonadota* at all stages of wastewater treatment plants [85], which could indicate that sewage sludge can introduce or enrich taxa from this group. In addition, Meng et al. 2019 have reported that *Pseudomonadota* could be an indicator of non-mature compost [86], which may help explain the high basal respiration and bacterial growth in soils with sludge compared with plant compost soils (Fig. 1). For its part, *Actinomycetota*, are more specialized in degrading hardly biodegradable waste such as cellulose [87, 88], and had a greater contribution in N metabolism, especially in soils with COVG. Therefore, the application of organic amendments could contribute to a microbiome effective in making up for the nutrients associated with the type of soil organic matter provided, especially nitrogen (N) and phosphorus (P) [75]. Concretely, NMDS ordination plots (Fig. S2b and S2b), PERMANOVA analyses (Additional file 1 and 2) showed clear changes in bacterial community genus level composition in relation to organic amendment treatments suggesting that the bacterial abundance was greater in the amended plots than in the unamended ones (Fig. S2c and S4c). This would agree with the higher content of bacterial fatty acids detected and higher basal respiration, suggesting enhanced bacterial growth and activity, even higher than Natural soils especially in soils containing SS and blends of SS with plant compost. In contrast, in soils amended with vegetable compost (COVG and COHort), these parameters were similar to Natural soils (Fig. 1). Sludge inputs would be promoting the biodegradation of soil organic matter and increasing microbial activity and enzyme synthesis [89, 90]. In addition, in our previous studies the molecular organic matter characterization revealed a higher labile compounds content in soil with sludge, such as carbohydrates of low molecular weight as well as higher mineralization rate [11, 43]. In contrast, vegetable composts provided a more recalcitrant organic matter with a higher content of compounds derived from lignin and cellulose [11]. In addition, although all organic amendments mainly provided nitrogen compounds [11], and TN content increased significantly compared to unamended soils, their highest values were found in the soils modified with sludge and its mixtures. Different authors have emphasized that organic amendments from sewage sludge enrich the soil in nitrogen compounds, especially when they were thermally dried [91, 92]. In addition, the organic amendments influence was most evident in taxa present in processes associated with N metabolism, in which potential taxa groups associated with soils treated with each organic amendments and untreated soils were clearly differentiated. However, taxa involved in processes linked to P turnover in phosphonates metabolism showed a homogeneous distribution, without a clear differentiation between groups (Fig. 6). Some authors have described similar patterns, highlighting a functional convergence in taxa involved in phosphorus turnover [93–95]. The high diversity of genera involved in many of the P functions found here, although not surprising due to the universal need for P in cellular life [96], also highlights that further MGS analysis of P functions in complex environments is warranted compared to the relatively more studied N cycle functions in relevant organisms. Further highlighting this dearth of knowledge was the greater occurrence among P contributors of genera that did not meet the CoverageChecker thresholds (Fig. 8). This indicates either that these P functions actually belong to many novel genera within the same bacterial families (implying more characterization of environmental strains is needed) or that the lower overall RPKM contributions of the P functions (compared to N) make achieving the 1% coverage or 50 confirmed reads cutoffs difficult, even if the assignments are taxonomically correct and those genera are present. We suspect it is somewhat of a combination of both phenomena, as some of the genera that failed validation at 1% coverage did have coverages at the > 0.1% threshold (equivalent to mapping 3 kb of a 3 Mb genome), indicating trace amounts present, but which we conservatively cannot deny could be misassignments by Kraken2.

The main potential contribution of organic amendments to the processes detected in N metabolism was ammonium assimilation. This is a key step that allows microorganisms to incorporate NH_4_^+^ into organic compounds, playing a fundamental role in nitrogen cycling and microbial growth [97]. Two main pathways facilitated this process: the glutamate dehydrogenase pathway (*gdhA*) (GDH) [98], and the glutamine synthetase-glutamate synthetase pathway (GS-GOGAT) (*glnA*, *gltB* and *gltD* genes) [99], the latter being, the predominant across all analyzed soils, highlighting its key role in regulating the conversion of inorganic N to NH_4_^+​^ and maintaining nitrogen balance in microbial communities [100]. This finding aligns with previous studies indicating that the GS-GOGAT pathway is the primary mechanism for ammonium assimilation under low NH_4_^+ ​^availability due to its high affinity for ammonium and energy efficiency [101]. The abundance of N assimilation genes, such as *glnA* and *gltBD* increased significantly in soil amended with gardening compost (COVG first, and COHort second), as well as their mixtures with SS compared with the rest of the treated soils. In addition, the abundance of *glnA* was similar to Natural reference soils, and the urease activity was also higher in these treatments, suggesting a greater potential for NH₄⁺ production. Since urease can generate NH₄⁺ from urea, this may have facilitated greater ammonium assimilation through *glnA* [102]. This was corroborated by the positive correlation observed of *glnA* and *gtlS* (genes involved glutamate metabolism) with urease activity and pH, indicating that the plant compost treatment provides a greater amount of nutrients for plants during restoration. Liang et al. (2024) found that genes involved in ammonia assimilation, such as *glnA*, correlated positively with NH₄⁺-N and pH and reported that the availability of ammonium activates the ammonia assimilation network [103]. In fact, the vegetable compost contributed to proliferation of bacterial genera such as *Streptomyces* which prefer ammonium as their primary nitrogen source [104], and this genus exhibited the highest potential contribution to ammonium assimilation in the studied soils (Fig. 4). Other genera, as *Nocardioides*,* Bradyrhizobium* and *Mesorhizobium* were also present in the soil and possess the genetic potential to carry out ammonium assimilation [105, 106]. This suggests a degree of functional redundancy within the microbial community [107], where multiple taxa contribute to the same nitrogen assimilation process, potentially buffering the system against shifts in microbial composition due to changes in nitrogen availability.

In contrast, soils amended with sewage sludge (SS) exhibited the lowest *glnA* contribution and urease activity, likely due to higher ammonium (NH₄⁺) and others inorganic nitrogen inputs compared to plant-based amendments and unamended soils, as supported by total nitrogen content (TN) measured across treatments (Fig. 1). However, SS treatments and their mixtures presented the highest values of potential contribution of genes related to denitrification (such as *nosZ*, *nirKS*, *norBC* and *napAB*), and nitrification (*hao*,* pmoABC-amoABC*), indicating a greater potential dynamism in the nitrogen cycle compared to soils with plant compost amendments and unamended soils. In addition, SS-treated soils, could provide favorable conditions for denitrification and the proliferation of denitrifying bacteria such as *Pseudomonas* [108]. Among denitrifying bacteria, the genus *Pseudomonas* is recognized as one of the most dominant groups in soils worldwide [108]. Rodríguez-Berbel et al. (2021) reported the promotion of this taxon after the application of sewage sludge in semi-arid climate soils [15]. In other studies, *Pseudomonas* spp. have demonstrated the ability to solubilize phosphate, fix nitrogen, and produce indole acetic acid - key factors that contribute to plant growth promotion and soil fertility improvements [109]. Likewise, sludge amendments promoted the proliferation of *Nitrosospira*, which had an important potential contribution in nitrification processes. This pattern may be associated with a higher availability of nitrogenous substrates in these soils, since SS showed the highest TN content, which is consistent with the potential presence of greater ammonium (NH₄⁺) concentrations (although NH₄⁺ was not measured), and such conditions could be linked to an increased potential for nitrification processes [110]. During this process, ammonia-oxidizing bacteria (AOB), such as *Nitrosospira*, catalyze the oxidation of NH₄⁺ into nitrite (NO_2_^−^) and subsequently into nitrate (NO_3_^−^) [110]. Previous studies have demonstrated that *Nitrosospira* predominates in ammonia oxidation, particularly in soils with relatively high ammonium concentrations [111, 112]. This supports our findings, where a positive correlation was observed between the nitrification process and total nitrogen content. Moreover, the resulting nitrate can then serve as an electron acceptor in denitrification [113]. Denitrification is a microbially driven process in which nitrate and nitrite are sequentially reduced to nitric oxide (NO), nitrous oxide (N₂O), and molecular nitrogen (N₂), playing a crucial role in nitrogen cycle dynamics [114]. The abundance of key denitrifying genes (*nirK*,S napAB and *nosZ*) increased significantly following the application of organic amendments, particularly in soils amended with SS and CoHort. This increase can be attributed to the substantial enhancement of soil organic carbon (TOC) and total nitrogen (TN) induced by organic amendments, and especially in soils with of SS application, a trend also reported by Dong et al. (2022) [115]. Previous research has demonstrated that pH influences the abundance of denitrification-related genes, with *nirK* displaying a positive correlation with pH [116]. On the contrary, in our study, a positive correlation between denitrification-associated genes and pH was not found. These results suggest that the addition of nitrogen could increase microorganisms with the potential to carry out denitrification processes, thus stimulating the nitrogen cycle in the soil. Despite the fact that sewage sludge can provide beneficial bacteria in restoration processes, their key role in denitrification processes can promote the loss of nitrogen compounds from the soil in the form of nitrogenous gases in soils [117], with consequent negative feedback for climate change. The lower potential contribution of vegetable compost to the denitrification process could indicate a shift toward nitrogen retention in the soil, through the potential transformation of highly mobile nitrates into ammonium that could be assimilated by plants and may be associated to reducing nitrogen losses in the soil [102, 118]. The combination of treatments generated a more balanced environment, which could potentially promote both denitrification and nitrification, which indicate the potential for organic matter mineralization. This suggests that the mixtures may result in intermediate functional potentials, reflecting contributions from both types of amendments. This pattern may indicate a broader representation of nitrogen-cycling genes, consistent with the presence of taxa capable of inorganic N acquisition under low NH₄⁺ conditions, possibly influenced by the labile carbon provided by the sludge [100, 102].

In the other hand, the highest abundances of *narGZHYIV* genes, which encode key enzymes involved in dissimilatory nitrate reduction to ammonium (DNRA), were detected in amended soils. DNRA is an important, but relatively understudied pathway in soil nitrogen cycling [119]. Studies have also revealed a surprisingly high prevalence of bacteria carrying the genetic components required for DNRA [120]. Recent research has highlighted its widespread occurrence across various terrestrial ecosystems, including forests, grasslands, croplands, rice paddies, and deserts [120–123]. These amendments likely contributed to the development of a diverse microbial community capable of facilitating DNRA, indicating a greater functional potential for this pathway in the modified soils.

Organic amendments had a significant effect on the increase of organic matter, as well as available phosphorus (P) in treated soils compared to Natural soils and unamended Controls soils. Organic amendments provide a significant contribution to the P supply [124], given that organic matter input can have feedback in P turnover [125]. In calcareous soils, such as those analyzed in this study, P availability is particularly low due to strong interactions between phosphate ions and calcium, resulting in the formation of insoluble compounds that restrict its bioavailability [126], and organic amendments application could improve the P bioavailability [127]. In this context, microorganisms have evolved mechanisms to acquire and metabolize less accessible forms of organic P, such as phosphonates, allowing them to survive in environments with limited P availability [128].

Moreover, the naturally low organic matter content, high pH and the absence of rainfall in semiarid areas lead to microbial metabolic constraints, including P turnover [129, 130], highlighting the importance of exploring alternative microbial pathways, such as phosphonate utilization, as a potential source of bioavailable P [131, 132].

The detailed visual representation of the potential functions identified in phosphonate and phosphinate metabolism (Fig. 4), allowed us to observe a complex network of enzymatic reactions, highlighting the diversity of biochemical processes involved in the biosynthesis, degradation, and regulation of these compounds. However, the phosphonates contain a highly stable C–P bond that requires specialized enzymes for cleavage and certain bacteria taxa have evolved metabolic strategies to use these compounds as nutrients sources [133]. In fact, several metabolic pathways for phosphonate degradation have been identified, with the C–P lyase pathway being the most extensively studied to date [26]. In effect, organic amendments mainly were associated with an increased potential degradation of phosphonates through this pathway, which could allow bacterial communities to obtain phosphorus for growth from the phosphonates [133]. The higher abundance of genes such as *phnG*,* phnH*,* phnI*,* phnJ*,* phnL*, and *phnM* are essential for the C–P lyase cleavage activity [134]. However, *phnJ* clearly stood out as the most abundant potential gene in soils with amendments, having a significantly greater contribution in soils modified with COHort and mixtures of amendments (Fig. 7). The rest of the genes were also present in unamended Control and Natural soils, and sometimes with similar contributions to those in amended soils, which could be attributed to the phosphorus limitations in the edaphic environment mentioned above. In addition, this pattern may suggest greater availability or accessibility of organic phosphonates in amended soils, which can serve as substrates for microbes capable of cleaving C-P bonds. In 2023, Ruffolo et al. analyzed bacterial genomes in the Integrated Microbial Genomes and Microbiomes database of the Joint Genome Institute and identified *phnJ* in more genomes than any other C–P lyase gene, with the majority of these homologs belonging to classes within the phylum *Pseudomonadota* [84]. These findings align with our results, where *phnJ* contributed 41% of the total RPKMs, and *Pseudomonadota* was the phylum with the highest potential contribution to phosphonate metabolism, along with *Actinomycetota*. Organic amendments also increase microbial diversity and activity, which was associated with higher relative abundance of bacteria harboring *phnJ*, such as strains of the genus *Pseudomonas* that have been evaluated as inoculants to promote plant growth by solubilizing inorganic phosphates [135–137]. In addition, these amendments are known to improve soil conditions by enhancing microbial respiration and nutrient cycling [15, 58, 138], which may provide favorable conditions for phosphonate-utilizing microbes a competitive advantage, particularly under low inorganic P availability. Several studies have used strains of the genus *Pseudomonas* as bacterial inoculants to promote plant growth by solubilizing inorganic phosphates [135–137]. These microorganisms perform several key functions such as mineralization, solubilization, and plant-microorganism interactions that contribute significantly to the maintenance of soil fertility and healthy plant growth [16].

Additionally, alternative pathways distinct from the classical C–P lyase pathway have been studied, showing independence from Pi regulation and a preference for biogenic phosphonates [139, 140]. Enzymes such as 2-AEP: pyruvate aminotransferase (EC 2.6.1.37) encoded by the *phnW* gene [141], and the phosphonoacetaldehyde hydrolase product of *phnX* (phosphonatase; EC 3.11.1.1) [142], are part of these alternative routes for the assimilation of phosphonates. Genes associated with these pathways were detected in the metagenomic dataset, indicating a potentially diverse repertoire of microbial strategies for phosphonate metabolism in these soils.

Several limitations should be considered when interpreting these findings. Functional interpretations are based on metagenomic gene abundances and therefore do not reflect actual process rates, microbial activity, or gene expression. Key nitrogen and phosphorus transformations were not directly measured, and neither ammonium concentrations nor nitrogen losses were quantified, limiting inferences about nutrient dynamics. Moreover, the responses described correspond to six months after amendment application in a restored quarry soil and may differ under other environmental conditions or timescales. Despite these limitations, the results provide valuable insight into how organic amendments shape microbial communities and their functional potential in degraded semiarid soils.

## Conclusion

The organic amendments not only altered the physical and chemical properties of the Technosols, but also reflected shifts in the bacterial communities and their functional potential. Overall terms, the organic amendments were linked to the proliferation of specific microbial communities contributing to the metabolism of P and N, even after the relatively short term (6 months). The taxonomy of these communities varied depending on the chemical composition of the different organic amendments and differed from the non-amended soils. In addition, the functional redundancy observed in both metabolisms suggests that several bacterial species can carry out similar processes, with adaptive specializations that are influenced by the specific characteristics of the applied treatment. Sludge-amended soils showed higher relative abundance of genes associated with mineralization and denitrification processes, while vegetable composts, rich in resilient compounds, displayed higher relative abundances of genes involved in ammonium assimilation and phosphonate degradation processes. The combination of sewage sludge and vegetable compost showed an intermediate functional potential between the amendments that composed it, and displayed intermediate gene profiles involved in N and P cycling, suggesting a potential contribution to nutrient recycling in the restored mining soils. This initial exploration provides baseline insights into taxonomy-function relationships, although many novel taxa remain to be investigated, which could help compare the general effects on N cycling and P turnover during the restoration of soils in other studies and contexts. However, these results should be treated with caution, and longer-term monitoring would be useful to investigate how the interactions between soil properties and the taxonomy-function relationships in the biogeochemical processes of nutrients in soils evolve, to better understand the sustainability and suitability of treatments with organic amendments in the restoration of semi-arid mining soils. Additionally, monitoring the effects of organic amendments in this sense could provide valuable information on the dynamics of MGS metabolic profiles over multiple timescales, and deserves additional attention in microbial and soil ecology in restored soils.

## Supplementary Information

Below is the link to the electronic supplementary material.

Supplementary Material 1

Supplementary Material 2

Supplementary Material 3

Supplementary Material 4

Supplementary Material 5

## Data Availability

The authors declare that the data supporting the findings of this study are available within the paper and its Supplementary Information files and in public repositories. Raw stratified RPKM files for both the N and P turnover are available in Additional files 1+2; CoverageChecker collated results at the genus-level and full raw results are available as Additional files 3+4; and the original raw metagenomic sequencing data are available at the ENA under accession number PRJEB47869. The bio-informatic pipeline for Quality-controlled reads con be found at: [https://github.com/LangilleLab/microbiome_helper/wiki/Metagenomics-Standard-Operating-Procedure-v3] . All codes for Taxa-function visualizations are available at: [https://github.com/dhwanidesai/JarrVis] . In addition, the CoverageChecker v0.0.1-beta tool is available at: [https://github.com/R-Wright-1/genome_coverage_checker/] . KrakenTools script can be found at: [https://github.com/jenniferlu717/KrakenTools] .

## Abbreviations

COHort: 100% vegetable compost made from greenhouse horticultural crop residues
COHort + SS: 50:50 mixture of COHort + SS
CON: Experimental plots without addition of organic amendments used as control
COVG: 100% vegetable compost made from garden waste
COVG + SS: 50:50 mixture of COVG + SS
DNA: Deoxyribonucleic acid
EC number: Enzyme Commission number
KEGG: Kyoto Encyclopedia of Genes and Genomes
MGS: Metagenomic sequencing
NADP+: Nicotinamide adenine dinucleotide phosphate
NADPH: Nicotinamide adenine dinucleotide phosphate hydrogen
NAT: Areas close to the experimental plots, but undisturbed by mining activities, selected as quality reference “natural” soils
rRNA: Ribosomal ribonucleic acid
RPKM: Reads Per Kilobase Million
SS: Sewage sludge residues treated by mesophilic digestion, thermal dehydration at 70 °C and centrifugation
