# Supplementary material for: Linking microbial taxonomy and function in N and P metabolism: a study of organic amendments in semiarid restored soils
Source: Environ Microbiome. 2026 Jan 8;21:23. doi: 10.1186/s40793-025-00845-9 (PMC12874732; doi:10.1186/s40793-025-00845-9)
Supplement: Supplementary file 5 — Supplementary Material 5 [file 40793_2025_845_MOESM5_ESM.docx]

Supplementary figures


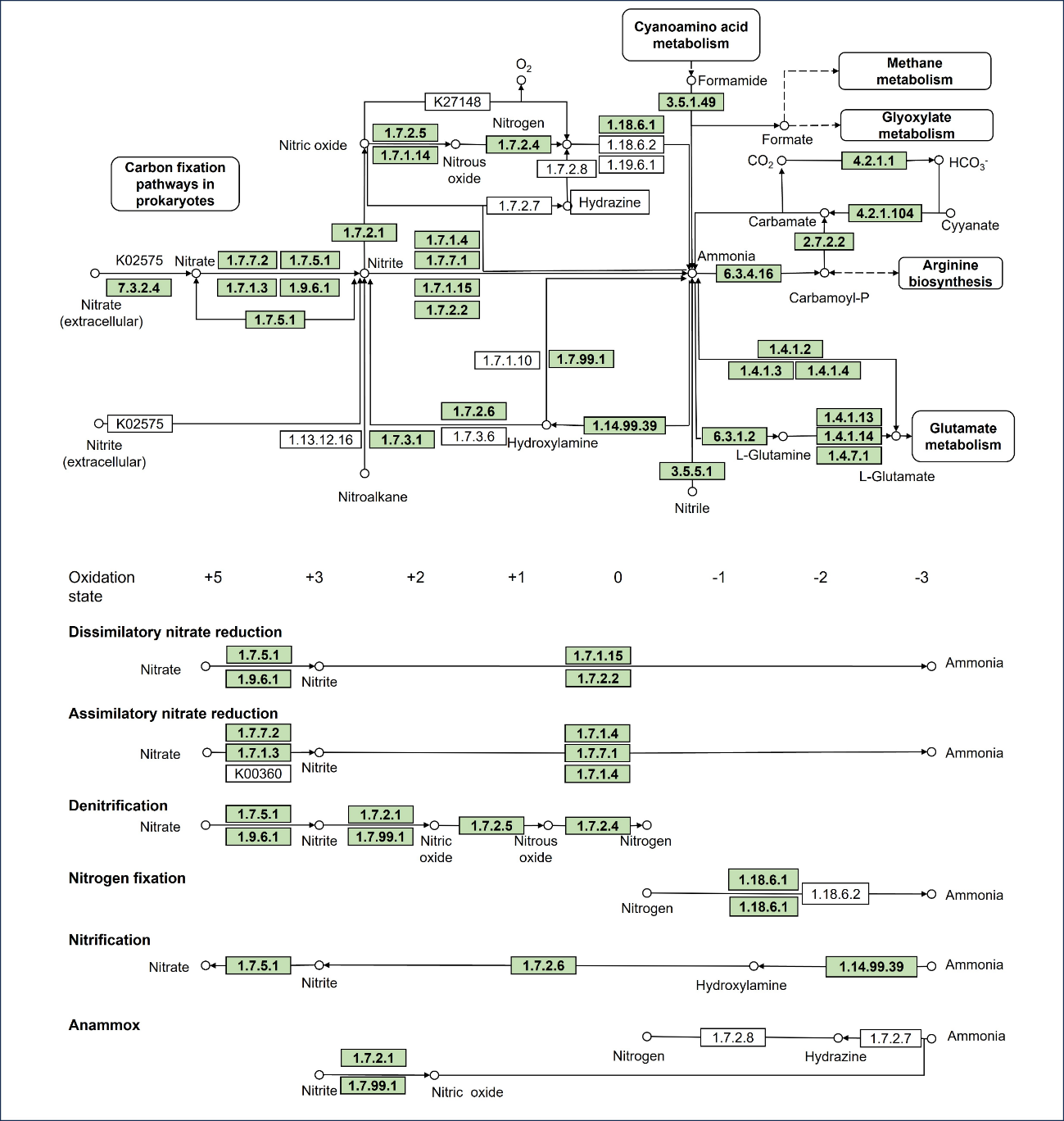


**Figure S1.** Mapping of potential KEGG pathway N metabolism functions in treatments. Green boxes mark potential functions identified in at least one treatment.


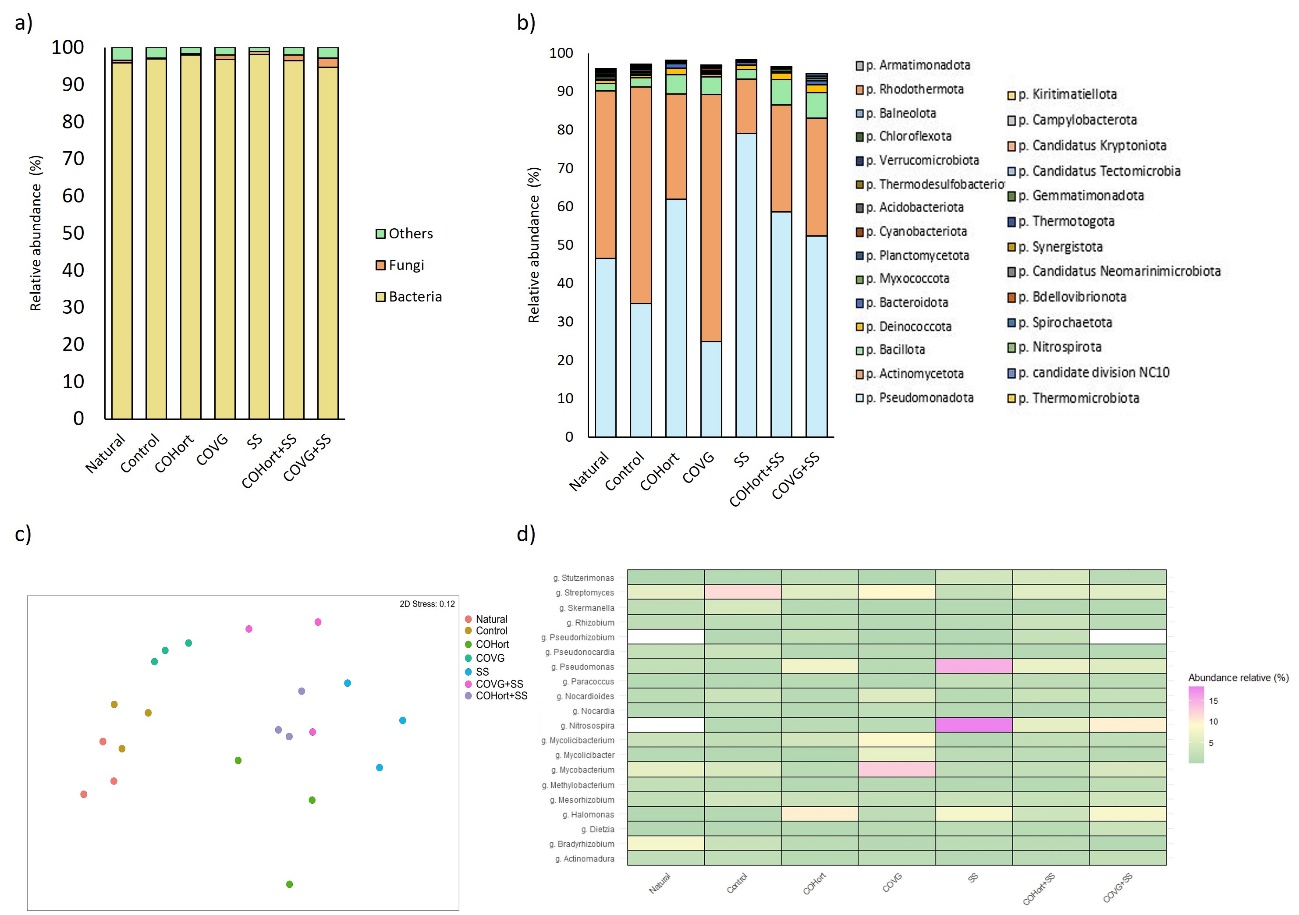


**Figure S2.** Taxonomic composition of microbial communities involved in soil nitrogen cycle at the domain (a) and phylum (b) levels in amended and unamended soils, as identified through shotgun metagenomics. (c) NMDS ordination of soil samples based on the taxonomic structure of their microbial communities at the genus level. (d) Heatmap displaying the relative abundance of the top 20 genera.


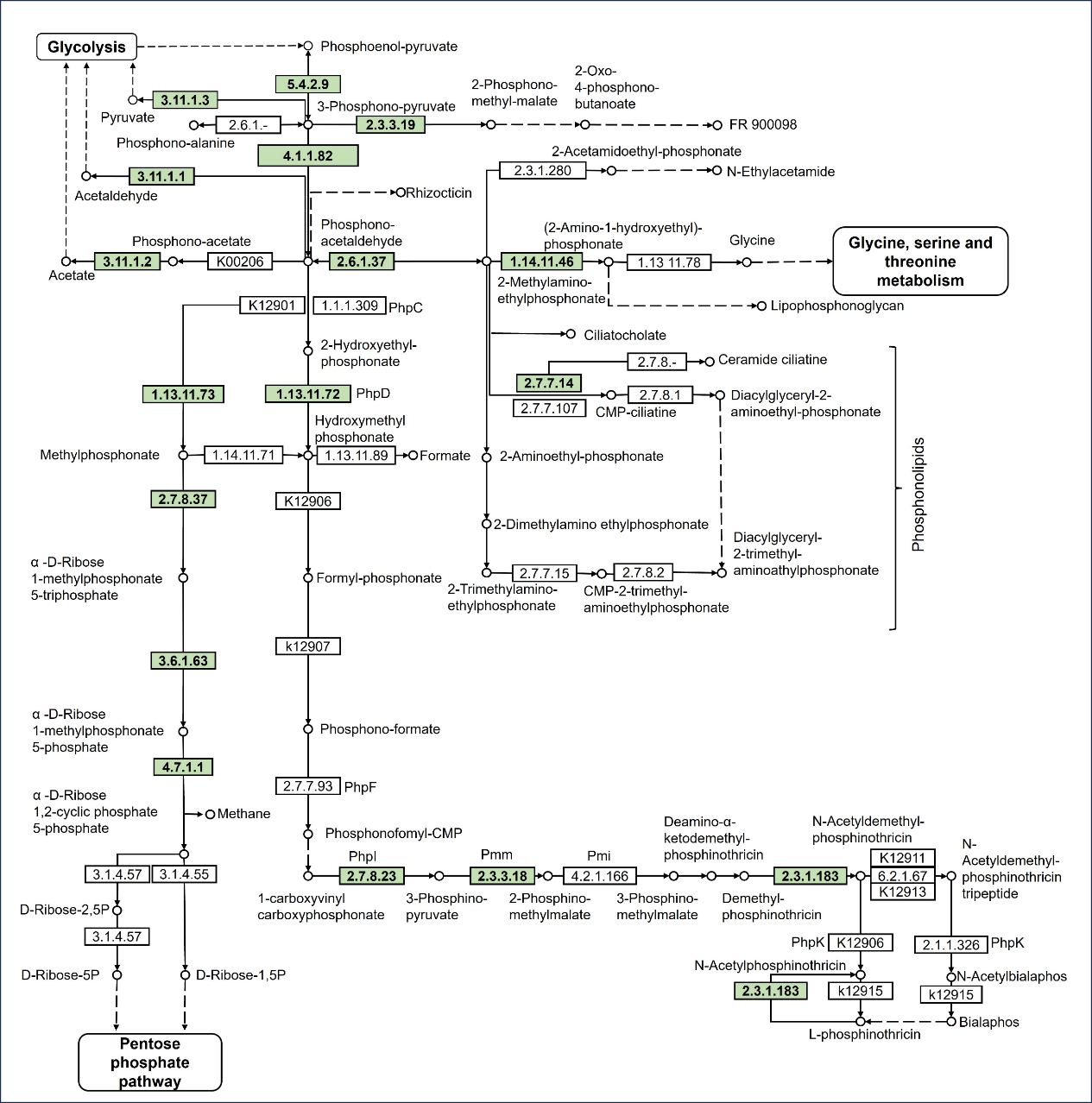
**Figure S3.** Mapping of potential KEGG pathway P metabolism functions in treatments. Green boxes mark potential functions identified in at least one treatment.


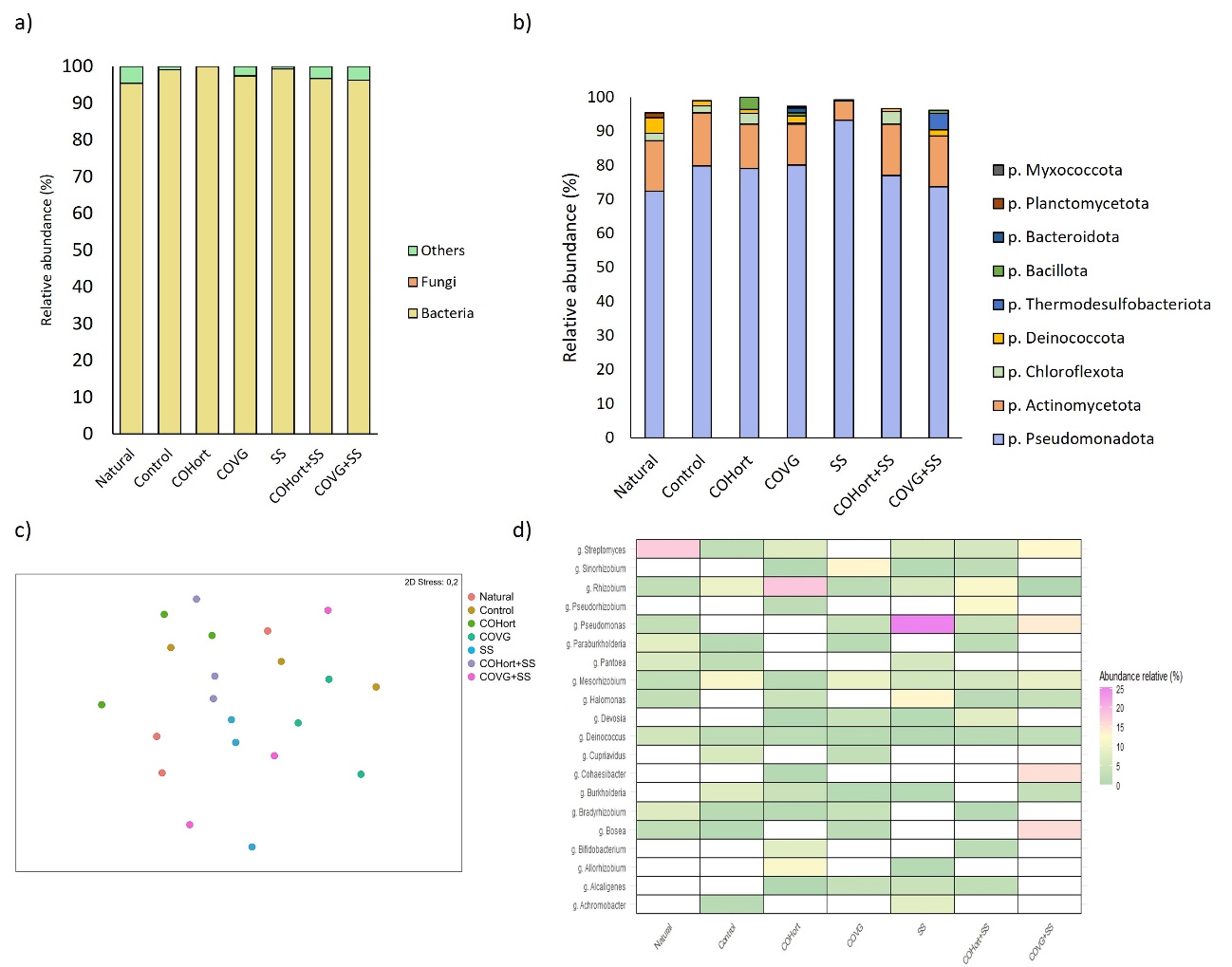


**Figure S4.** Taxonomic composition of microbial communities involved in soil phosphonate and phosphinate metabolism at the domain (a) and phylum (b) level in amended and unamended soils, identified by shotgun metagenomics. (c) NMDS ordination of soil samples based on the taxonomic structure of their microbial communities at the genus level. (d) Heat map showing the relative abundance of the top 20 genera.
